# A General Strategy for Nanohybrids Synthesis via Coupled Competitive Reactions Controlled in a Hybrid Process

**DOI:** 10.1038/srep09189

**Published:** 2015-03-30

**Authors:** Rongming Wang, Wantai Yang, Yuanjun Song, Xiaomiao Shen, Junmei Wang, Xiaodi Zhong, Shuai Li, Yujun Song

**Affiliations:** 1 Department of Physics, School of Mathematics and Physics, University of Science & Technology Beijing, Beijing 100083, China; 2 College of Materials and Engineering, Beijing University of Chemical Technology, Beijing 100029, China; 3 School of Materials Science and Engineering, Beihang University, Beijing 100191, China

## Abstract

A new methodology based on core alloying and shell gradient-doping are developed for the synthesis of nanohybrids, realized by coupled competitive reactions, or sequenced reducing-nucleation and co-precipitation reaction of mixed metal salts in a microfluidic and batch-cooling process. The latent time of nucleation and the growth of nanohybrids can be well controlled due to the formation of controllable intermediates in the coupled competitive reactions. Thus, spatiotemporal-resolved synthesis can be realized by the hybrid process, which enables us to investigate nanohybrid formation at each stage through their solution color changes and TEM images. By adjusting the bi-channel solvents and kinetic parameters of each stage, the primary components of alloyed cores and the second components of transition metal doping ZnO or Al_2_O_3_ as surface coatings can be successively formed. The core alloying and shell gradient-doping strategy can efficiently eliminate the crystal lattice mismatch in different components. Consequently, varieties of gradient core-shell nanohybrids can be synthesized using CoM, FeM, AuM, AgM (M = Zn or Al) alloys as cores and transition metal gradient-doping ZnO or Al_2_O_3_ as shells, endowing these nanohybrids with unique magnetic and optical properties (e.g., high temperature ferromagnetic property and enhanced blue emission).

Composition and strucrue controlled hybridization has become an effective approach for realizing multi-functionality, stability and/or producing new physicochemical properties of nanomaterials due to the surface and interface enhanced properties and the synergistic effect among components^1^,^2^,^3^,^4^,^5^,^6^,^7^,^8^,^9^,^10^,^11^,^12^,^13^,^14^,^15^,^16^,^17^,^18^,^19^,^20^,^21^,^22^,^23^,^24^. With the advancement of science and technology promoted by nanohybrids, large scale, controlled-engineering of nanoparticles (NPs) becomes more and more urgent to fulfill their academic and industrial perspectives^3^,^10^,^25^,^26^,^27^,^28^,^29^,^30^,^31^,^32^. Controlled successive coating on preformed cores is one simple and effective method, but still challenging, particularly for ultra-small nanohybrids with surface coatings that are incompatible with cores (e.g., metal-dielectrics or metal-semiconductors) due to their crystal lattice mismatching and large curvatures^33^. Besides large-scale ability, spatiotemporally thermal-dynamic control of each stage in the nanohybrids formation is required to optimize their compositions and structures. Traditional batch-wise techniques have enabled the synthesis of varieties of NPs^10^,^15^,^33^,^34^,^35^. However, They are usually limited by their low productivities and/or difficulty in spatiotemporally splitting the NP formation stages.

Microfluidic approaches have been attracting more and more attention in the NPs synthesis due to their reduced scaling up risk and the precise reaction kinetic control of each stage along the microchannel that is desired for sketching formation mechanism spatiotemporally for controlled morphology and structure^9^,^17^,^26^,^27^,^28^,^29^,^30^,^36^,^37^,^38^,^39^,^40^. Recently, a great progress has been achieved by Erdem et al. in microfluidic synthesis of NPs by developing a silicon-chip-based two-phase flow microreactor with multiple thermally-isolated zones^41^. This chip-based microreactor enables the separation of nucleation and growth during nanoparticle formation using TiO_2_ NPs as model. We also invented polymer-chip-based microfluidic reactors, which can realize controlled growth of NPs by freezing the NPs solution in the microchannel with a low-temprature quenching solution^27^,^30^,^42^,^43^. In spite of complicated fabrication process, these chip-based microreactors are capable of layer-by-layer assembly, representing a milestone in the controlled scale-out synthesis of nanoparticles. We further developed simple programmed microfluidic processes (SPMPs), which have shown great powers in spatiotemporally splitting the NP formation and scale-out features^16^,^43^. Herein, we extend SPMPs to a sequenced microfluidic and batch-cooling process with stage-controlled reaction kinetics. Growth of nanoparticles can be well-terminated at the defined stage by rapid cooling the reaction solution from the microchannel in the collecting receiver with steeply reduced temperature. This hybrid process was then used in the structure and composition controlled synthesis of nanohybrids by coupling competitive reactions, or sequenced reducing-nucleation and co-precipitation, which were realized by introducing a second metal salt with low electrochemical potential (i.e., AlCl_3_, ZnCl_2_) into the primary-metal-salt solution.

Similar as our previous results^16^,^43^, the latent time of nucleation in the coupled competitive reactions and the growth of nanoparticles can be well-controlled due to the formation of controllable intermediates. Thus, spatiotemporal-resolved synthesis can be realized by this hybrid process, which enables us to investigate nanohybrid formation at each stage through their solution color changes and TEM images for optimizing their compositions and structures. By adjusting the bi-channel solvents and kinetic parameters (e.g., reactant concentrations, flow rates and reaction temperatures) at each stage, the primary components with alloyed cores and the second components with the primary metal gradient-doping ZnO or Al_2_O_3_ as surface coatings can be successively formed. The implied alloying and gradient-doping strategy can efficiently eliminate the crystal lattice mismatch in core and shell components. Consequently, ultra-small magnetic-dielectrics (e.g., FeAl@Al_(1-x)_Fe_x_O_y_), magnetic-semiconductors (e.g., CoZn@Zn_(1-x)_Co_x_O_y_), plasmon-dielectrics (e.g., AgAl@Al_(1-x)_Ag_x_O_y_) and plasmon-semiconductors (e.g., AuZn@Zn_(1-x)_Au_x_O_y_) NPs of unique magnetic and optical properties can be synthesized using N-methyl-2-pyrrolidone (NMP) and/or water as bi-channel solvents at a wide range of flow rates.

## Results

Figure s1 in supporting information (SI) shows the typical experiment setup of the sequenced microfluidic and batch-cooling process^16^,^43^. This setup includes: one syringe pump for the metal-salt solution (e.g., FeCl_2_ and/or CoCl_2_ in NMP mixed with polyvinylpyrrolidone (PVP)) (1); one syringe pump for the reducing-agent solution (e.g., NaBH_4_ in NMP) (2); two preheating stainless steel spirals (3–4) immersed in the thermostatic tank 1 (inner diameter (ID) = 127 μm, length (L) = 15 cm) for the heating of the metal-salt solution and the reducing-agent solution from 20°C to 200°C; one Y-mixer (ID = 250 μm or more, L = 4 ~ 5 mm) for the reactants to react to form precursors and then to initiate nucleation (5); one microtubing spiral (ID = 127 ~ 500 μm, L = 10 ~ 60 cm or more) in the thermostatic tank 2 with temperature from 20°C to 200°C for finishing nucleation and growth of nanoparticles (6); and one product receiver (7) protected by inert gas (i.e., N_2_), with temperature controlled from −15°C to 200°C by a chiller and/or an oil heating bath in the thermostatic tank 3. The heating process of tank 1, tank 2 and tank 3 is controlled by the oil bath with the boiling point of oil about 260°C. Therefore, the temperature range for reaction can be extended up to 200°C using high boiling temperature solvents (e.g., NMP). The residence time in tank 1 and tank 2 can be conveniently conrolled by the flow rate, the length and diameter of the microchannel. The mean residence time in tank 3 depends on the flow rate and the reactant volume in the syringe. For example, the mean residence time is about 10 mins if the flow rate is controlled at 1.0 mL/min for each syringe pump and the reactant solution in each syringe is 20 mL. The temperature in the tank 3 is usually controlled no more than 15°C to freeze the growth of nanoparticles for desired sizes and undesired Ostwald ripening.

To realize multi-functionalization and/or robust surface protection in single NPs, alloyed core-shell NPs or oligomer NPs with alloy cores and surface oxide coatings are expected to work^9^,^10^,^19^,^23^,^30^. However, the surface coating of materials different from cores usually is very challenging by conventional epitaxial growth, particularly for those with crystal lattices mismatching with the cores. To achieve this aim, a new synthesis strategy, or *in-situ* reduction of mixed-metal ions and precipitation of second-metal oxides by forming alloys as cores and doping/mixed oxides as shells or surface-coatings, was developed by directly introducing the second-metal-salts with low standard electrode potentials (i.e., Zn^2+^: V_0_ = −0.763 V; Al^3+^: V_0_ = −1.662 V) into the primary-metal-salt solutions (i.e., CoCl_2_ and/or FeCl_2_, HAuCl_4_, AgNO_3_). According to their standard electrode potentials, the primary-metal salts will be reduced into atoms more easily and quickly than the second-metal salts. Simutaneously, nucleation of these atoms into nanocrystals will form a competitive process with the co-precipitation of these metal ions to form metal hydroxides and then metal oxides. The former process is usually a little bit faster than the later. As a result, the preformed tiny Co, Fe, or CoFe, Au, Ag nanocrystallites after nucleation firstly will be surrounded by the second-metal ions. Since the reduction of the second-metal ions into atoms cannot be ruled out, these atoms will be alloyed into these nanocrystallites, forming alloy cores. Along with the reduction reaction, the pH value of the reaction solution changes, leading to the acceleration of co-precipitation of these metal ions into hydroxide species. Surfaces of these preformed alloy nanocrystallites (i.e., Zn or Al alloyed Co, Fe Au, Ag or CoFe NPs) can perform as nucleation sites for the precipitation reactions, consequently forming mixed metal hydroxide coatings on these alloy cores^44^,^45^. These metal hydroxides can be further transferred into metal oxides by dehydration in the post treatment, forming oxide shells or coatings. Some of the primary- or second-metal atoms on surfaces or near surfaces of the alloy cores can be redoxed or even directly collided into the oxide shells or coatings to form the metal atom/ion doping passivation layer^46^,^47^. In addition, some primary-metal ions and reducing agents can be included into the second-metal hydroxides during the co-precipitation, finally forming the second metal oxide coatings doped by the primary metal. The primary or the second metal ions can be reduced into atoms by the reducing agents included in the coatings and the reduced second metal atoms in the surface coatings can also reduce the primary metal ions into atoms. These metal atoms may gradually diffuse from the surface coatings into the cores, which can increase the core size slightly. With the growth of particles and the reform of the surface coatings during the collecting stage in the cooling reciever, the above effects will lead to the formation of the primary metal cores alloyed by the second metal atoms and the second metal oxide shells gradient-doped by the primary metal atoms/ions.

This strategy is demonstrated by the synthesis of magnetic-dielectrics (MAl@Al_(1-x)_M_x_O_y_) and magnetic-semiconductors (MZn@Zn_(1-x)_M_x_O_y_) nanohybrids (M = Co, Fe, Ag, Au, etc.) as models by introducing the competitive reducing-nucleation and precipitation reaction using all NMP-phased reaction systems, or NMP as bi-channel solvents. Similar as in the case of CoFe NPs^43^, the formation process of these nanohybrids can also be spatiotemporally divided into 4 stages. The first stage is the formation of organometallic intermediates by the reduction reaction. The primary metal salts and some of the second metal salts in NMP are reduced into metal atoms by NaBH_4_ (rx-1). These metal atoms can be coordinated by the 5-membered cyclic lactam in PVP and/or NMP to form kinetic stable organometallic intermediates (rx-2) before nucleation. Simultaneously, Zn^2+^ and Al^3+^ ions can react with NaBH_4_ to form relatively stable zinc or aluminum borohydrides (rx-4, rx-5), which can decompose either to metal hydrides rapidly (rx-6, rx-7) or to metallic atoms (zinc or aluminum, rx-8 and rx-9) stabilized by the 5-membered cyclic lactams, similar as rx-3^46^,^47^. The second stage is the nucleation stage by the competitive reducing-nucleation and precipitation. These kinetic stable intermediates will nucleate to form tiny nanocrystallites after the kinetic parameter is changed (e.g., elevated temperature or proton solvents H_2_O) (rx-3). These zinc or aluminum atoms can mix with those primary-metal atoms if a transient nucleation occurs, leading to the formation of alloyed-metal nuclei. Depending on the solution pH value, the formed second metal hydrides can either reduce the primary metal ions (e.g., Fe^2+^ or Co^2+^) into atoms or re-decompose into metal ions (rx-10 and rx-11) that will form metal hydroxides (rx-12 and rx-13) surrounding or even directly precipitating on the alloyed-metal nuclei surfaces^44^,^45^. Note: the primarily formed surface coatings are not complete or very loose. The third stage is the growth stage: the alloyed metal nuclei with partial coatings or loose shells will grow up to form final nanohybrids. These metal atoms formed in the previous 2 stages can be stabilized by PVP and NMP again (rx-2), and then be absorbed by the coated nuclei, and finally be recrystallized into the alloyed cores or traped in the coatings through diffusion. Simultaneously, the formed-metal hydroxides continue depositing on the surface of the preformed alloyed nuclei to increase the coating thickness that will gradually increase the diffusion resist of atoms or ions between surface coatings and cores. Consequently, nanohybrids formed by alloy cores and the second metal oxides gradient-doped by the primary metal atoms/ions as shells can be synthesized, whose growth can be frozen or retarded in the temperature rapidly-reduced receiver during the 4^th^ termination stage. Clearly, these active metal atoms (Zn or Al) in the alloy cores can be used as sacrificed metal atoms to protect the oxidization of the primary-metal atoms by forming a thin layer of the second-metal oxides (ZnO or Al_2_O_3_). As a result, by adding ZnCl_2_ or AlCl_3_ into the primary-metal-salt solutions above, hybrid metal-dielectrics with the primary metal doping Al_2_O_3_ as coatings or hybrid metal-semiconductors with the primary metal doping ZnO as coatings can be obtained, respectively. By this alloying and gradient-doping strategy, lattice mismatch between cores and shells can be eliminated efficiently.
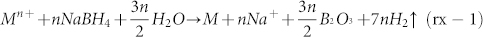

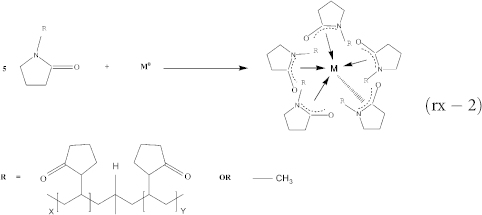


Similar as in the case of CoFe NPs, these new reaction systems to synthesize nanohybrids by competitive reduction and precipitation of mixed metal salts are often accompanied by color changes, such as the formation of grey or white organometallic intermediates (rx-2, rx-3). Most important, the stability of these intermediates can be increased after adding the second metal salt into the primary metal salt solution, which makes it more convenient to identify each stage and optimize their kinetic parameters simply by color changes. Morphologies and structures of nanohybrids at different stages were characterized by TEM images, correlating with the reaction solution colors at different stages using the synthesis of magneto-dielectrics (i.e., FeAl@Al_(1-x)_Fe_x_O_y_ NPs) as example. The reaction solutions of formed intermediates, nucleation, growth, termination (collecting), Ostwald ripening and aggregation were obtained by tunning the residence time in the microchannel and the collecting time in the cooling receiver, which is controlled by channel lengths at a controlled reaction temperature and flow rate (details in Part I of SI).
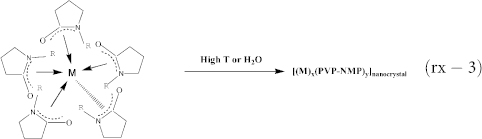

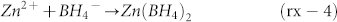

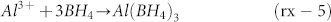

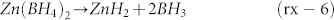

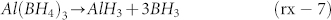

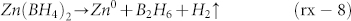

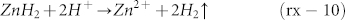

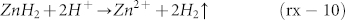

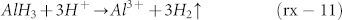

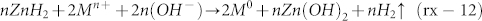

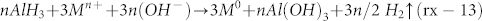


**Figure 1:**
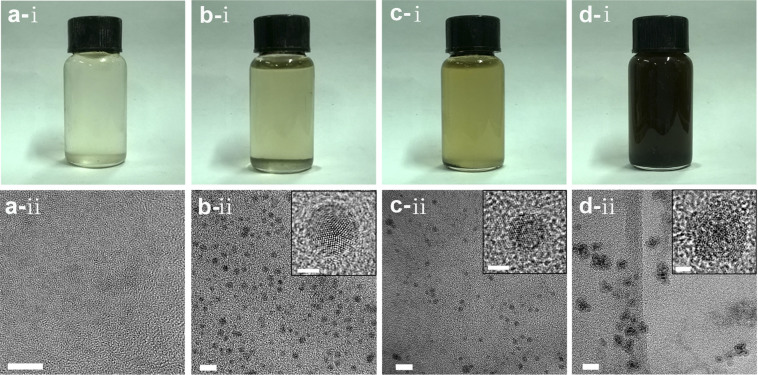
Reaction solution color change and TEM image of the resulted particle at different resident time in microfluidic channels after the reductant solution is mixed with the mixed metal salt solution using FeAl@Al_(1-x)_Fe_x_O_y_ (the FeCl_2_ and AlCl_3_ in NMP shows yellow color) as example. Color changes of the reaction solutions (a-i, b-i, c-i and d-i) and TEM images of the formed nanoparticle (a-ii, b-ii, c-ii, d-ii) after the reaction proceeds about 0.39 s (L_total_ = 10 cm), 0.44 s (L_total_ = 15 cm), 1.03 s (L_total_ = 35 cm) and 1.77 s (L_total_ = 60 cm), respectively. L_total_: the total microchannel length after the Y-mixer (5) in Fig. s1.

The mixed metal salt solution (FeCl_2_ and AlCl_3_) changes from yellow to white (Fig. 1a-i) as the reaction proceeds about 0.29 second (L_total_ = 10 cm) after it mixes with the reducing solution in the micro-mixer, indicating the formation of intermediates. As the intermediates were observed by TEM, most areas only show solute contaminating (local foggy areas) amorphous carbon film (Fig. 1a-ii). A few of nanocrystallinities (Fig. s2a) about 1.8 ± 0.4 nm (Fig. s2b) can be rarely found, which can be attributed to the localized initial nucleation due to the rapidly-increased local concentration by the solvent evaporation during the TEM sample preparation. The white intermediate solution will change to light-brown (Fig. 1b-i) and lots of highly-crystallized nanocrystals (Fig. 1b-ii) about 3.7 ± 0.6 nm (Fig. s2c) can be observed if the reaction proceeds for additional 0.15 second (L_total_ = 15 cm), indicating that the nucleation occurs. Since the nucleation is very fast, a slight growth of NPs in the microchannel and during the TEM sample preparation cannot be avoided. Thus, these nanoparticles are almost twice bigger than those observed in Figure 1a-ii and surface coatings can be clearly observed in many of them (inset in Fig. 1b-ii). The reaction solution becomes brown (Fig. 1c-i) as the reaction proceeds for additional 0.59 second (L_total_ = 35 cm). Interestingly, many of larger particles and lots of smaller particles can be observed (Fig. 1c-ii) in this stage. This is a common phenomena often observed during nanoparticle formation, where many larger particles will be formed by absorbing the solutes released from the smaller particles, or Ostwald ripening (OR), leading to a reduced mean diameter (3.1 ± 0.6 nm, Fig. s2d). The surface coatings of many NPs become thinner (inset in Fig. 1c-ii) than those NPs in Fig. 1b-ii. Clearly, these NPs preserve good crystalline cores but a little bit disordered surfaces, indicating that it is the surface coatings that firstly dissolve into the solution during OR, which can protect the cores from dissolving too fast. As the reaction proceeds for additional 1.33 seconds (L_total_ = 60 cm), the solution color becomes black (Fig. 1d-i). Surprisely, morphologies and crystal structures in NPs change dramatically (Fig. 1d-ii). Core shell morphology becomes more distinct, with cores of 3.1 ± 0.7 nm and the total diameter of 6.9 ± 2.5 nm (Fig. s2e, s2f). This result indicates that the elongated OR is mainly to increase the shell thickness (1.9 nm) since the core size changes little as comparing with that of the previous stage. Clearly, both shells and cores become amorphous (inset in Fig. 1d-ii) by comparing with those highly-crystallized NPs in the previous stages (Fig. 1b-ii and 1c-ii), indicating the reform of crystal structures both in cores and shells possibly due to the inclusion of metalloid boron and the doped second metal oxides (e.g., Al_2_FeO_4_) during OR. Therefore, termination of the growth at desired stage and the elimination of Ostwald ripening are very crucial in the morphology (size and surface coatings) and crystal structure control of nanohybrids. Otherwise, these NPs will experience Ostwald ripening where the random growth and reforming of surface coatings, interfaces and/or cores may occur, leading to broad size dispersion and/or structure change in each component.

**Figure 2:**
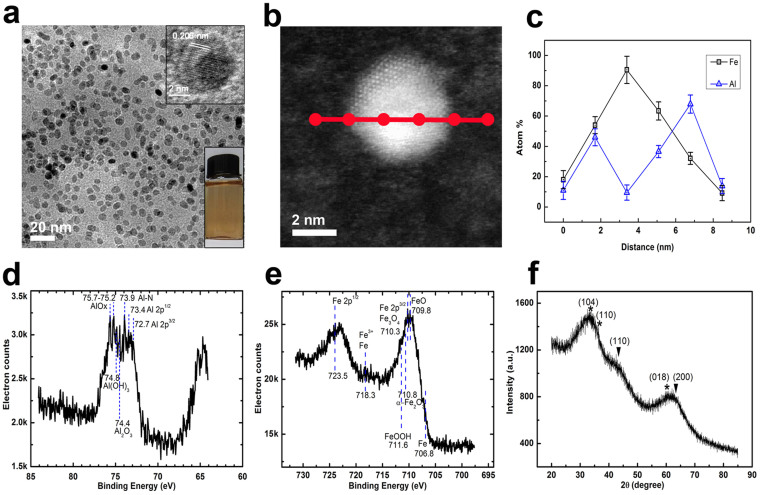
Wide-viewed TEM image (a), HR-TEM image of one single particle (a: inset), STEM-HAADF image of one single particle (b), point-by-point EDX scanning of one single particle (c), high energy resolution XPS for Al (d), high energy resolution XPS for Fe (e) and XRD (f) of FeAl@Al_(1-x)_Fe_x_O_y_ nanohybrids synthesized using all NMP-phased reaction systems at a resident time of 0.73 ~ 0.88 s in microfluidic channel and 20 min in the cooling receiver. Right-bottom inset in Fig. 2a is the NPs solution. JCPDS No for XRD analysis: bcc Fe, 6-0696; α-Al_2_O_3_, 46-1212; γ-Al_2_O_3_, 10-0425. In XRD (f), 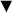: the corresponding metallic phases; *: the corresponding metal oxides.

After the systematical investigation of the reaction kinetics, nanohybrids with uniform morphology and good crystallinity can be obtained as the growth time in the microchannel is controlled in 0.30–0.45 second (L_total_ = 25–30 cm) and then the solution is collected in the cooling receiver (the resident time: ~20 min), where the light-brown solution becomes brown (Fig. 2a: right-bottom inset). The detailed reaction conditions are described in Part I of SI, together with those for other typical nanohybrids. As shown in Fig. 2a, uniform NPs with a mean diameter of 4.8 ± 0.3 nm (Fig. s3a-i) can be obtained. Even though the core shell structure is not so distinct, contrast differences between the inner parts and the surface layers can be observed from one typical HRTEM image (Fig. 2a: right-top inset; Fig. s4a) in spite of eccentric in shape. The STEM-HAADF (Z-contrast) images equipped with Gatan GIF 2000 energy filter system and energy dispersive X-ray spectrometries (EDX) for some typical particles were performed. Figure 2b is the STEM-HAADF image for one typical single particle, showing clear contrast difference between the center and the surface layer. The atom ratios between Fe and Al recorded by point-by-point EDX scanning (point step: ~1.6 nm) show a significant change from the surface layer to the center part of this particle (Fig. 2c). The Al/Fe ratio gradually increases from 10/90 at the center of the core (~2.5 nm in diameter) to 35/65 at the interface between the core and the surface coating (~1.0 nm thick), and to 50/50 ~ 70/30 at the out layer of the surface coating, forming an interface with gradually-decreasing Fe content.

The electronic structure of the consisting elements in the NPs can be well-defined by X-ray photoelectronic spectroscopy (XPS), giving further composition information of these NPs. The depth of the photoemitted electrons escaping from the very top surface of samples by the Al K_α_ X-ray source (1486.6 eV photons) is usually in the 0.5–3 nm range, comparable to half of the diameters of these NPs (~4.8 nm). Thereby, XPS can help us to obtain the electronic structures of elements in cores and coatings, or whether they are metallic or oxidized. The full XPS spectra for these samples are plotted in Fig. s5a. The full XPS spectra not only confirm the existence of Fe, Al and O, but also of C, B and N in the NPs. Carbon can be mainly from the stabilizer, or PVP. Boron can be possibly from the decomposition of some NaBH_4_ and the reducing reaction of rx-1, producing metallic boron that may be used as alloy element in metallic cores or B_2_O_3_ that may exist in the oxide coatings^17^. The nitrogen can be attributed from the stabilizer (i.e., PVP) and/or the formation of metal-nitrogen bonds as the intermediates decompose (rx-2 and rx-3).

The high resolution XPS spectrum for Al element (Fig. 2d) in FeAl@Al_(1-x)_Fe_x_O_y_ NPs shows two broad peaks centered at 73.2 eV and 75.2 eV, which can be indexed as the signals from Al 2p binding energy (BE) of metallic Al and Al 2p bonding energy of Al oxides. More precisely, shoulders at 72.7 eV and 73.4 eV can be indexed as 2p3/2 and 2p1/2 of metallic Al, respectively. The full XPS spectrum of FeAl@Al_(1-x)_Fe_x_O_y_ NPs also gives Al 1s binding energy of metallic Al, which can be attributed to the metallic cores. Several shoulders at 75.2–75.7 eV, 74.8 eV and 74.4 eV can be indexed as Al 2p bonding energies from AlO_x_, Al(OH)_3_ and Al_2_O_3_, respectively, which are doped by Fe in shells. Interestingly, there is a distinct shoulder at 73.9 eV that can be possibly indexed as Al 2p BE of Al-N bonds formed by the decomposition of Al-based intermediates (rx-3). The high energy resolution XPS spectrum for Fe element (Fig. 2e) in FeAl@Al_(1-x)_Fe_x_O_y_ NPs shows pronounced peaks at 710.3 eV and 723.3 eV, indexed as Fe 2p3/2 and Fe 2p1/2, which suggests that iron oxides are mainly of Fe_3_O_4_ that is mixed with Al_2_O_3_ in shells by checking the peak position of O1s (530.7 eV). A shoulder at 706.8 eV can be clearly observed from the asymmetry feature of the peak at 710.3 eV, which can be attributed to the BE of metallic Fe 2p3. Combination of the Fe 3s BE at 92.0 eV from the full XPS spectrum (Fig. s5a) of FeAl@Al_(1-x)_Fe_x_O_y_, the penetration depth of XPS (0.5–3.0 nm) and the size of FeAl@Al_(1-x)_Fe_x_O_y_ (4.8 nm), the existence of metallic iron in the FeAl cores of FeAl@Al_(1-x)_Fe_x_O_y_ can be reasonably confirmed by recalling the STEM-HAADF characterization. Additionally, existence of FeO and trace of α-Fe_2_O_3_ are possible acccording to the shoulder at 709.4 eV and the weak satellite peak at 718.3 eV.

The XRD pattern of these NPs (Fig. 2f) gives two broad peaks at 43.0° and 62.7° that represent the (110) plane and the (200) plane of bcc Fe, respectively, which is alloyed by Al and/or B according to the XPS analysis. Other three broad peaks/shoulders at 33.2° 36.0° and 60.3° represent the (104) plane, the (110) plane and the (018) plane of α-Al_2_O_3_, respectively. The Al_2_O_3_ layer should be doped by Fe and/or B according to the proposed formation mechanism, the above XPS and EDX characterization for the whole particles (Fig. s3a-ii).

According to the comprehensive analysis by HRTEM, STEM-HAADF, XPS, XRD and EDX, it can be reasonably concluded that cores in these NPs are made of iron-rich bcc FeAl alloys and surface coatings are mainly formed by Fe doped α-Al_2_O_3_ (e.g., Al_2_FeO_4_ or AlFeO_3_), some Fe doped Al(OH)_3_ coatings and trace of other kinds of FeO_x_ and AlO_x_. Interestingly, the Al/Fe ratio gradually increases from the inner part to the out surface, or forming a gradient doping metal-oxide shell. This kind of hybrid structures with gradient shells gives us a main reason that these NPs do not show a large crystal lattice mismatch and a distinct core-shell contrast. Using this method, other magneto-dielectric nanohybrids, such as CoFeAl@Al_(1-x)_(CoFe)_x_O_y_ and CoAl@Al_(1-x)_Co_x_O_y_ can be synthesized, having the similar alloyed cores and gradient-doping metal-oxide shells.

**Figure 3:**
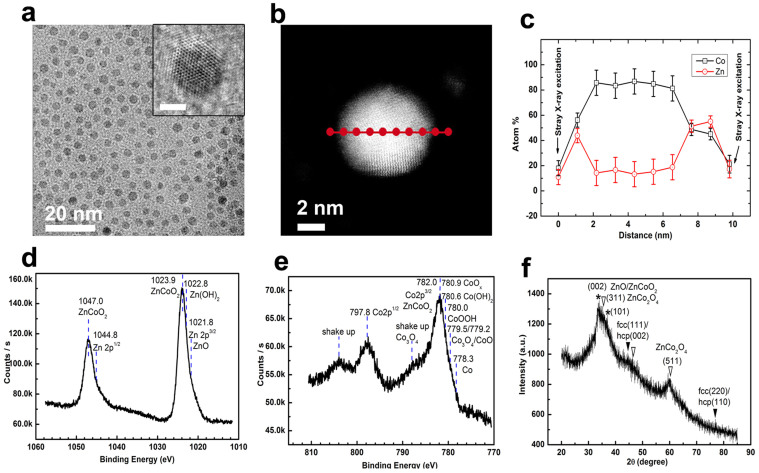
Wide-viewed TEM image (a), HR-TEM image of one single particle(a: inset, scale bar: 2 nm), STEM-HAADF image of one single particle (b), point-by-point EDX scanning of one single particle (c), high energy resolution XPS for Zn (d), high energy resolution XPS for Co (e) and XRD (f) of CoZn@Zn_(1-x)_Co_x_O_y_ nanohybrids synthesized using all NMP-phased reaction systems. JCPDS No for XRD analysis: fcc Co, 15-0806; hcp Co, 05-0727; wurtzite ZnO, 36-1451; spinel ZnCo_2_O_4_, 23-1390. In XRD (vi), 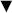: the corresponding metallic phases; 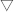 and *: the corresponding metal oxides.

Encouraged by this success, we further extend this method in other kind of nanohybrids to test its generality, such as magneto-semiconductor nanohybrids (i.e., CoZn@Zn_(1-x)_Co_x_O_y_). Figure 3a gives one wide-viewed TEM image of the as-synthesized CoZn@Zn_(1-x)_Co_x_O_y_ nanohybrids, showing uniform dispersed size distribution. Their size statistics give a mean diameter of 3.5 ± 0.2 nm (Fig. s3b-i). HRTEM image (Fig. 3a: inset) for one typical single nanoparticle shows the distinct contrast and crystal lattice differences between the surface layer and the inner part (Fig. s3b). The STEM-HAADF image (Fig. 3b) for one single CoZn@Zn_(1-x)_Co_x_O_y_ particle suggests the discernable brightness contrast between the center and the surface in spite of not so sharp. The Co/Zn atom ratios recorded by point-by-point EDX scanning show a significant change from the out surface to the center of this particle (Fig. 3c), indicating a Co core slightly alloyed by Zn and a ZnO shell heavily doped by Co by considering the formation mechanism and EDX for the whole particles (Fig. s3b-ii). Interestingly, the Zn/Co ratio is gradually increased from the core edge (2.5 ~ 3.0 nm in diameter) to the out surface of the shell (1.5–2 nm thick), finally reaching a Zn/Co atomic ratio of about 1:1.

The full XPS spectrum for CoZn@Zn_(1-x)_Co_x_O_y_ NPs is plotted in Figure s5b, confirming the existence of Co, Zn, O, C, B and N in these NPs, which can be attributed to the similar sources as in FeAl@Al_(1-x)_Fe_x_O_y_ NPs. The peak at 1023.3 eV (blueshift from the peak at 1021.7 eV for Zn 2p3/2) in the high resolution XPS of Zn (Fig. 3d) and the peak at 782.0 eV (blue shift from 780.5 for Co 2p3/2) in the high resolution XPS of Co (Fig. 3e) indicate the existence of Co doping ZnO. Both ZnCoO_2_ and ZnCo_2_O_4_ can exist in CoZn@Zn_(1-x)_Co_x_O_y_ NPs according to the STEM-HAADF-EDX analysis and the distinct shake up peaks in the high resolution XPS of Co. The asymmetry feature (low energy tails in each peak) in Figure 3d and the possible sub-peak at 778.3 eV in Figure 3e suggest existence of metallic Zn and Co, which can be mainly attributed from CoZn alloy cores. According to the penetration depth of XPS on these compounds and results from the HRTEM and STEM-HAADF-EDX analysis, CoZn@Zn_(1-x)_Co_x_O_y_ NPs should be formed by the metallic Zn alloyed Co cores and Co/Co^2+^/Co^3+^ gradient-doping ZnO surface coatings. In the out surface of the coatings, there possibly exist some CoOOH, Co(OH)_2_ and Zn(OH)_2_ by considering the above analysis and the proposed mechanism (existence of H_2_O in the base reaction environments). The possible sub-peaks from 779.2 eV to 780.9 eV suggest existence of varieties of cobalt oxides and cobalt hydroxides that can form Co/Co^2+^/Co^3+^ doped ZnO and Zn(OH)_2_.

The XRD pattern of these NPs (Fig. 3f) gives two broad or weak peaks at 44.5° and 70.0° that can be indexed from the (111) plane and the (220) plane of fcc Co or the (002) plane and the (110) plane of hcp Co, respectively, which is alloyed by Zn and/or B according to the XPS analysis and peak shifts. Other two broad peaks and shoulders at 33.8°, 35.3°, 36.9°, 46.4°, 59.9° represent the (002) plane of wurtzite ZnO (PDF 36-1451) possibly doped by Co^2+^/Co (ZnCoO_2_/ZnCoO), the (311) plane of cubic ZnCo_2_O_4_, the (101) plane of wurtzite ZnO possibly doped by Co^2+^/Co, the (400) plane and the (511) plane of cubic ZnCo_2_O_4_, respectively, which may be mixed by B_2_O_3_ according to the peak shifts, the proposed formation mechanism and the XPS and EDX characterization of the whole particles (Fig. s3b-ii). Recalling the STEM and XPS analysis, the XRD results suggest the existence of ZnCo_2_O_4_ at out surface layer and ZnCoO/ZnCoO_2_ at sub-surface layer of the metal oxide shell.

According to the above analysis by HRTEM, STEM-HAADF-EDX, XPS, XRD and EDX, it can be reasonably concluded that cores in these NPs are made of cobalt-rich fcc/hcp CoZn alloys and surface coatings are mainly formed by Co/Co^2+^ doped ZnO (e.g., ZnCoO or ZnCoO_2_) and ZnCo_2_O_4_, some Co/Co^2+^/Co^3+^ doped Zn(OH)_2_, some CoOOH and Co(OH)_2_, and/or some B_2_O_3_ inclusion. Similar as FeAl@Al_(1-x)_Fe_x_O_y_, a Co/Co^2+^/Co^3+^ gradient-doping ZnO shell is formed.

As using all NMP-phased reaction systems, it was found that the latent time for the intermediates to nucleate became so long that the reaction had to be controlled at very low flow rates (usually ≤1.0 mL/min) if the reaction temperature was controlled below 90°C and the primary-metal-salt concentrations were controlled below 50 mmol/L. Otherwise, long tube is required so that more energy and construction materials are needed. The enhanced stability of intermediates, possibly due to the ion effects from the addition of the second-metal-salt (i.e., ZnCl_2_ or AlCl_3_), is not desired for high productivity even though it provides additional control time for nucleation. Starting investigation of the difference in reactivity between dry and hydrated metal salts and sequential bottle-batch experiments, we found that water could significantly enhance the formation of NPs by a mechanism of accelerating intermediate decomposition (rx-3) for rapid nucleation. This eventually directs toward developing another reaction system based on NMP-phased reductants (A, Fig. s1) and aqueous-phased metal salts (B, Fig. s1). Rapid reaction after mixing A and B enables to increase flow rates (e.g., from 0.8 ~ 1.0 mL/min to 3.0 ~ 5.0 mL/min or higher) for narrow nucleation stage, which is of great benefit for high productivity.

**Figure 4:**
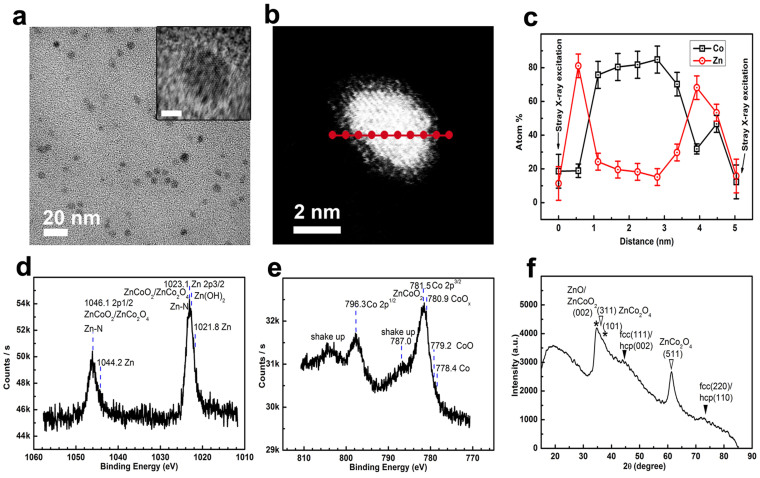
Wide-viewed TEM image (a), HR-TEM image of one single particle (a: inset, scale bar: 2 nm), STEM-HAADF image of one single particle (b), point-by-point EDX scanning of one single particle (c), high energy resolution XPS for Zn (d), high energy resolution XPS for Co (e) and XRD (f) of CoZn@Zn_(1-x)_Co_x_O_y_ nanohybrids synthesized using aqueous-phased metal salt reaction systems. JCPDS No for XRD analysis: fcc Co, 15-0806; hcp Co, 05-0727; wurtzite ZnO, 36-1451; spinel ZnCo_2_O_4_, 23-1390. In XRD (vi), 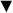: the corresponding metallic phases; 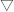 and *: the corresponding metal oxides.

By this new reaction system, one magneto-semiconductor nanohybrid (i.e., CoZn@Zn_(1-x)_Co_x_O_y_) was firstly synthesized at a high flow rate of 3.0 mL/min as model (detailed synthesis condition in Part I of SI and Table 1). Figure 4a is the TEM image of CoZn@Zn_(1-x)_Co_x_O_y_ NPs synthesized by the aqueous-phased metal salt process, suggesting a narrow size distribution. Statistics on their diameters give a mean diameter of 4.4 ± 0.4 nm (Fig. s3c-i). The core shell contrast between the inner part and the surface layer can be clearly observed from the HRTEM image for one typical single particle (inset in Fig. 3a, Fig. s4c). The STEM-HAADF image (Fig. 4b) for one single CoZn@Zn_(1-x)_Co_x_O_y_ particle suggests a gradually-changed contrast from the inner part to the out surface. Similar as those synthesized by all NMP-phased process, analysis on the element content changes from the inner part to the surface (Fig. 4c) implys a Zn alloyed Co-rich core and a Co-doping ZnO shell by considering the formation mechanism and EDX result for the whole particles (Fig. s3c-ii).

The full XPS spectrum for CoZn@Zn_(1-x)_Co_x_O_y_ NPs (Fig. s5c) confirms the existence of Co, Zn, O, C, B and N in these NPs. The high-resolution XPS spectrum for Zn in CoZn@Zn_(1-x)_Co_x_O_y_ nanohybrids (Fig. 4d) also shows two asymmetric peaks centered at 1023.1 eV and 1046.1 eV, which can be indexed as Zn 2p3/2 and Zn 2p1/2, respectively. The blueshift peak at 1023.1 eV by comparing with the peak at 1021.7 eV for Zn 2p3/2 and the blue shift Co 2p3/2 peak at 781.5 eV (from 780.5 eV) in the high energy resolution XPS of Co (Fig. 4e) indicate the existence of Co/Co^2+^ doped ZnO, such as ZnCoO or ZnCoO_2_. The two distinct shake up peaks at the high-energy side of Co 2p3/2 and Co 2p1/2 peaks indicate that most of cobalt in Zn_(1−x)_Co_x_O_y_ is bivalent, or mainly ZnCoO_2_. However, ZnCo_2_O_4_ cannot be excluded according to the XRD characterization (Fig. 4f). The asymmetric features of the BE peaks for Zn and Co also suggest existence of metallic Zn (BE: 1021.8 eV and 1044.2 eV), metallic Co (BE: 778.4 eV), Zn(OH) and Zn-N bonds, CoO and Co(OH)_2_. According to the HRTEM and STEM characterization, these metallic metals can be mainly from the Zn alloyed Co cores and those compounds can be only from the Co-doping ZnO coatings.

The XRD pattern of these NPs (Fig. 4f) shows similar but more distinct featured peaks than those CoZn@Zn_(1-x)_Co_x_O_y_ NPs synthesized by all NMP-phased process, possibly due to the increased size. Two broad and weak peaks at 44.5° and 72.5° represent the (111) plane and the (220) plane of fcc Co or the (002) plane and the (110) plane of hcp Co, respectively. Other two broad peaks and shoulders at 34.7°, 36.0°, 37.1° and 61.4° represent the (002) plane of wurtzite ZnO possibly doped by Co^2+^/Co (ZnCoO_2_/ZnCoO), the (311) plane of cubic ZnCo_2_O_4_, the (101) plane of wurtzite ZnO possibly doped by Co^2+^/Co and the (511) plane of cubic ZnCo_2_O_4_, respectively, according to the EDX characterization (Fig. s3c-ii) for the whole particles and the formation mechanism. By recalling the STEM-HADDF and XPS analysis, XRD results indicate the existence of ZnCo_2_O_4_ at outermost layers and ZnCoO/ZnCoO_2_ at sub-layers of the metal oxide coating.

Similar as those CoZn@Zn_(1-x)_Co_x_O_y_ synthesized by all NMP-phased reaction process, it can be reasonably concluded that these NPs are made of cobalt-rich fcc/hcp CoZn alloys as cores, and of Co/Co^2+^ doped ZnO (e.g., ZnCoO or ZnCoO_2_) and ZnCo_2_O_4_ as surface coatings according to the above analysis by TEM, HRTEM, STEM-HADDF, XPS, XRD and EDX. The shell is made of a Co/Co^2+^/Co^3+^ gradient-doping ZnO surface coating. In addition, some Co/Co^2+^/Co^3+^ doped Zn(OH)_2_ or CoOOH and Co(OH)_2_ and some B_2_O_3_ may be included in the shell and the metallic alloy core may include some B.

Similar as the all NMP-phased reaction systems, the new aqueous-phased metal salt reaction system also shows its generality by the successful synthesis of other nanohybrids with alloyed cores and gradient-doping shells at a high flow rate no less than 3.0 mL/min, such as CoAl@Al_(1-x)_Co_x_O_y_, FeAl@Al_(1-x)_Fe_x_O_y_, FeZn@Zn_(1-x)_Fe_x_O_y_ (x < 1). Realization of gradient nanohybrids using aqueous-phased metal salts boosts our faith to extend our strategy into other kinds of nanohybrids synthesis that may have more challenge to overcome crystal lattice mismatch, such as plasmonic-dielectric (AgAl@Al_(1-x)_Ag_x_O_y_) or plasmonic-semiconductor (AuZn@Zn_(1-x)_Au_x_O_y_) nanomaterials.

**Figure 5:**
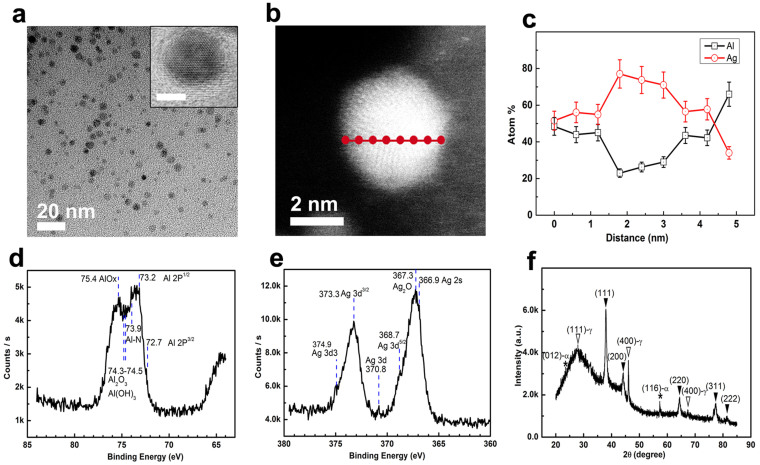
Wide-viewed TEM image (a), HR-TEM image of one single particle (a: inset, scale bar: 2 nm), STEM-HAADF image of one single particle (b), point-by-point EDX scanning of one single particle (c), high energy resolution XPS for Al (d), high energy resolution XPS for Ag (e) and XRD (f) for AgAl@Al_(1-x)_Ag_x_O_y_ nanohybrids synthesized using aqueous-phased metal salt reaction systems. JCPDS No for XRD analysis: fcc Ag, 15-0806; fcc Al, 04–0787; α-Al_2_O_3_, 46-1212; γ-Al_2_O_3_, 10-0425;. In XRD (f), 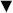: the corresponding metallic phases; 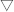 and *: the corresponding metal oxides.

Figure 5a show the TEM image of AgAl@Al_(1-x)_Ag_x_O_y_ NPs synthesized by aqueous-phased metal salt reaction system, suggesting a narrow size distribution. Statistics on their diameters give a mean diameter of 3.7 ± 0.3 nm (Fig. s3d-i). Their HRTEM images clearly show the highly-crystallized nature of these NPs with distinct core-shell morphology (inset: Fig. 5a) and crystal lattice difference (Fig. s4d). In order to further prove their core-shell structures, the STEM-HAADF image and point-by-point EDX characterization for some typical particles were performed. Figure 5b is the STEM-HAADF image of one single particle, which clearly showing the different contrast between the center and the surface layer of this particle. The element contents of Ag and Al by point-by-point EDX scanning (Fig. 5c) show a distinct gradient structure with decreased Ag/Al ratio from the center to the out surface of this particle. This result indicates a typical gradient core-shell structure: a Al alloying Ag core (about 75% Ag and 25% Al), an ~0.5 nm thick Al_2_O_3_ interlayer with Ag dopant gradually-decreasing from 75% to 50% and an ~1.2 nm thick Ag doping Al_2_O_3_ surface coating with the Ag/Al ratio kept at about 50%.

The full XPS spectrum for AgAl@Al_(1-x)_Ag_x_O_y_ nanohybrids (Fig. s5d) confirms the existence of Ag, Al, O, C, B and N in these NPs. Figure 5d is the high energy resolution XPS spectrum for Al in AgAl@Al_(1-x)_Ag_x_O_y_ nanohybrids, showing two distinct broad peaks with multi-shoulders. These peaks and shoulders can be indexed as the binding energy of metallic Al 2p3 at 72.7 eV, metallic Al 2p1 at 73.2 eV, Al-N at 73.9 eV, Al_2_O_3_ or Al(OH)_3_/AlOOH at 74.3–74.4 eV and AlO_x_ at 75.4 eV. The high energy resolution XPS spectrum for Ag in AgAl@Al_(1-x)_Ag_x_O_y_ nanohybrids (Fig. 5e) shows two pronunced peaks at 367.3 eV and 373.3 eV, which can be indexed as Ag 3d1/2 and Ag 3d3/2, respectively, indicating the existence of Ag_2_O in the NPs. The high energy resolution XPS spectrum for Ag also suggests existence of metallic Ag in the surface coatings of these NPs, as evidenced by the weak peak at 370.8 eV for Ag 3d and the shoulders at 374.9 eV for Ag 3d3, 368.7 eV for Ag 3d5/2 and 366.9 eV for Ag 2s. Recalling the STEM-HAADF image of one single particle and the EDX analysis for these NPs ensemble (Fig. s3d-ii), these results clearly suggest the gradient structure in these NPs, including Al alloyed metallic Ag cores, the Ag/Ag^+^ doped Al_2_O_3_ interlayer with the Ag/Ag^+^ dopant decreasing from the core to the surface coating, and the AlAgO_2_ at the outmost surface, which can be further confirmed by XRD analysis (Fig. 5f). Of course, the existence of Ag doping Al_2_O_3_ near the metallic AgAl cores cannot be excluded due to the sharply increased Ag content.

Their XRD peaks (Fig. 5f) at 38.0°, 44.1°, 64.4°, 77.4° and 81.6° show slight variation from those of the standard fcc Ag and fcc Al. They can be reasonably indexed as the inflection from the (111), (200), (220), (311) and (222) planes of Al alloyed fcc Ag, respectively, according to XPS analysis. Peaks at 32.5°, 46.1° and 67.4° can be indexed as the (111), (220) and (400) planes of Ag doping γ-Al_2_O_3_, peaks at 23.3° and 57.3° can be indexed as the (012) and (116) planes of Ag doping rhombohedral α-Al_2_O_3_ and the broad peak at 27.9° can be indexed from amorphous Al(OH)_3_/AlOOH by combination of the above XPS analysis. Therefore, it can be reasonably concluded that these nanohybrids are made of AgAl alloy as cores, Ag/Ag^+^ gradient-doping γ-/α-Al_2_O_3_ and some amorphous Al(OH)_3_/AlOOH as surface coatings according to the XRD, XPS, STEM-HAADF analysis and the EDX for the whole NPs (Fig. s3d-ii).

**Figure 6:**
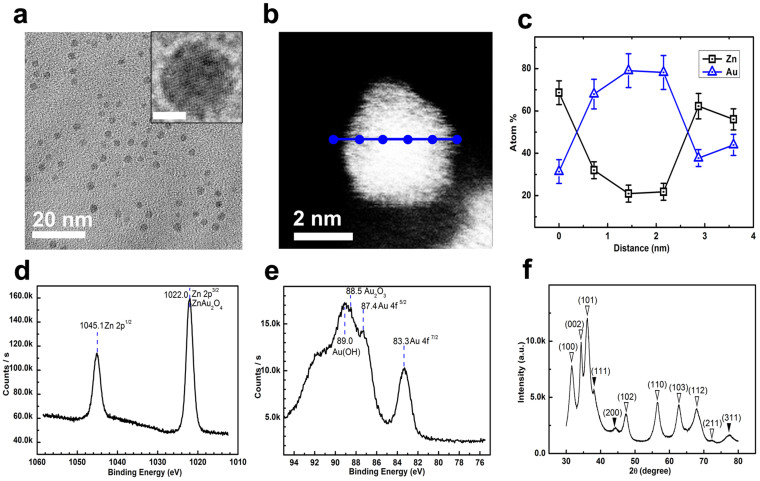
Wide-viewed TEM image (a), HR-TEM image of one single particle (a: inset, scale bar: 2 nm), STEM-HAADF image of one single particle (b), point-by-point EDX scanning of one single particle (c), high energy resolution XPS for Zn (d), high energy resolution XPS for Au (e) and XRD (f) for AuZn@Zn_(1-x)_Au_x_O_y_ nanohybrids synthesized using aqueous-phased metal slat reaction systems. JCPDS No for XRD analysis: fcc Au, 05-0727; wurtzite ZnO, 36-1451. In XRD (f), 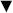: the corresponding metallic phases; 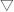: the corresponding metal oxides.

Figure 6a show the TEM image of AuZn@Zn_(1-x)_Au_x_O_y_ NPs synthesized by the aqueous-phased metal salt process, suggesting a uniform size distribution. Statistics on their diameters give a mean diameter of 3.6 ± 0.2 nm (Fig. s3e-i). Their HRTEM images clearly show the highly-crystallzed nature of these NPs with distinct core-shell morphology (Fig. 6a: inset) and crystal lattice difference (Fig. s4e). In order to confirm their core-shell structures, the STEM-HAADF image and point-by-point EDX characterization for some typical particles are performed. Figure 6b is the STEM-HAADF image of one single particle, which clearly shows the different contrast between the center and the surface layer of this particle. The element contents of Au and Zn recorded by point-by-point EDX scanning (Fig. 6c) show a distinct gradient structure with Au/Zn ratio decreasing from the core to the particle surface. This result indicates a typical gradient core-shell structure: a Zn alloying Au core (about 80% Au and 20% Zn in the core), an ~1.0 nm thick Au doped ZnO interlayer with Au dopant gradually-decreasing from 80% to ~30% and an ~0.5 nm thick Au doping ZnO surface coating with the Au/Zn ratio kept from 4/6 to 3/7.

Similar as other nanohybrids, the full XPS spectrum for AuZn@Zn_(1-x)_Au_x_O_y_ nanohybrids (Fig. s5e) confirms the existence of Au, Zn, O, C, B and N in these NPs. The high energy resolution XPS spectrum for Zn in AuZn@Zn_(1-x)_Au_x_O_y_ nanohybrids (Fig. 6d) shows two distinct peaks at 1021.8 eV and 1044.5 eV, representing Zn 2p3/2 and Zn 2p1/2. According to the symmetry peak feature and the peak shift to high energy, which are very different from those asymmetry feature in other nanohybrids (e.g., CoZn@Zn_(1-x)_Co_x_O_y_), ZnO doped by Au should be dominant in the top surface of these NPs. Only trace of metallic Zn exists in the surface coating according to the peak at 9.7 eV for Zn 3d binding energy and other peaks, such as Zn_loss_ binding energy at 102.9 eV, Zn 3s binding energy at 139.5 eV and Zn 2s binding energy at 1195.4 eV (Fig. s5e). The high energy resolution XPS spectrum for Au in AuZn@Zn_(1-x)_Au_x_O_y_ nanohybrids (Fig. 6e) gives two peaks at 83.3 eV and 87.4 eV, representing Au 4f7/2 and Au 4f5/2, respectively, which can be attributed from the metallic Au alloy cores. The other two shoulders at 88.5 eV and 89.0 eV in the high energy broad peak can be addressed as the binding energy for Au_2_O_3_ and the binding energy for Au(OH), respectively.

Distinct peaks (solid triangles) in their XRD pattern (Fig. 4b-v) at 38.1°, 44.2° and 77.3°, can be indexed as the reflection from the (111), (200) and (311) planes of Zn slightly-alloyed fcc Au, respectively, by recalling the above XPS analysis. Others strong peaks (void triangles) in their XRD pattern at 31.7°, 34.4°, 36.1°, 47.4°, 56.6°, 62.8°, 67.9° and 72.4° can be indexed as the reflections from the (100), (002), (101), (102), (110), (103), (112), (211) planes of wurtzite ZnO doped by Au/Au^3+^ according to the XPS analysis.

Combination of their TEM, HRTEM, STEM-HAADF, XPS and EDX analysis for the single NPs and NPs assemble (Fig. s3e-ii), it can be confidently deduced that cores of AuZn@Zn_(1-x)_Au_x_O_y_ nanohybrids are made of Zn slightly-alloyed Au and shells of AuZn@Zn_(1-x)_Au_x_O_y_ nanohybrids are mainly made of Au/Au^3+^ gradient-doping ZnO (e.g., ZnAuO, ZnAu_2_O_4_) possibly mixed with some Au(OH).

**Figure 7:**
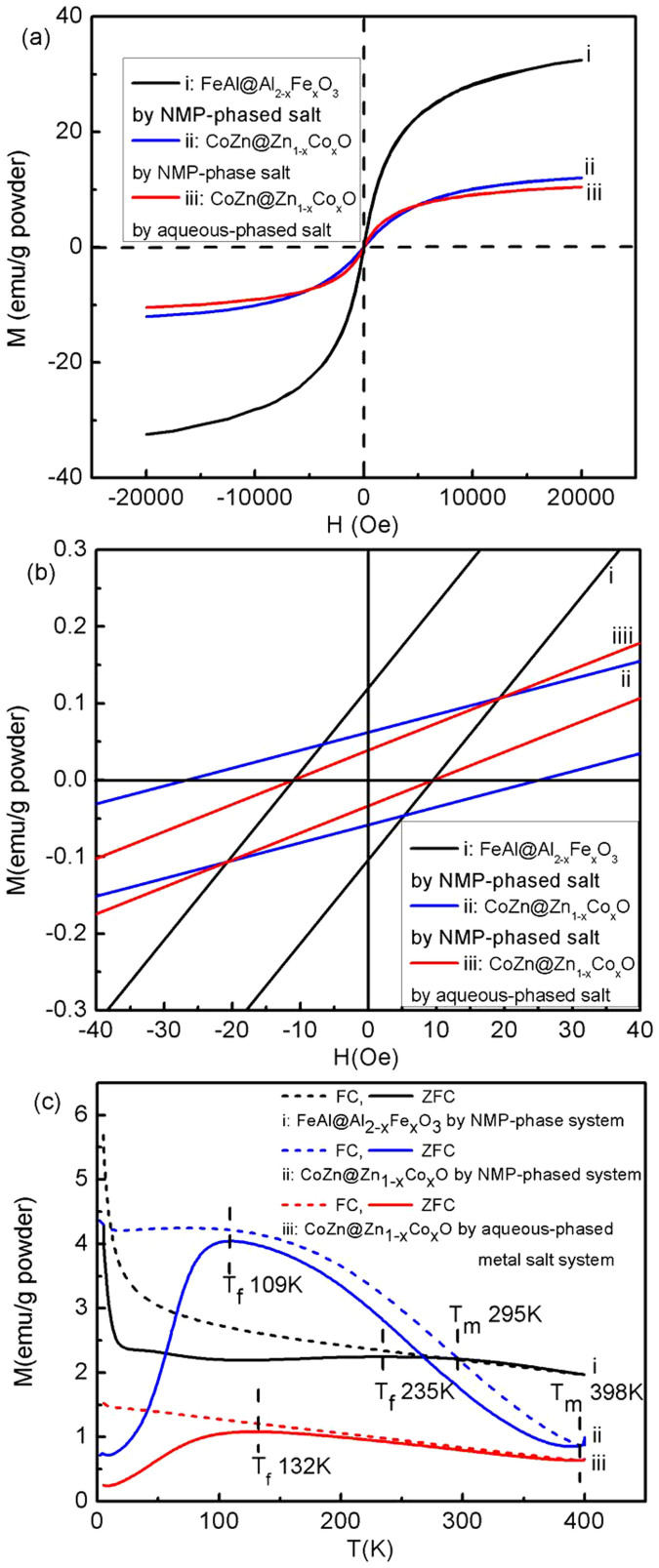
Full-range (a) and magnified-center (b) of room temperature hysteresis loop and the thermo-magnetism curves (c) (ZFC: zero-field cooling; FC: field cooling) of (i) FeAl@Al_(1-x)_Fe_x_O_y_ and (ii) CoZn@Zn_(1-x)_Co_x_O_y_ nanohybrids synthesized using all NMP-phased reaction systems and (iii) CoZn@Zn_(1-x)_Co_x_O_y_ nanohybrids synthesized using aqueous-phase metal salt reaction systems.

These nanohybrids with alloy cores and gradient shells preserve unique magnetic and optical peoperties. The room temperature hysteresis loops (RTHLs) and thermal-magnetism measurements (zero-field cooling (ZFC) and field-cooling (FC) curves with an applied field of 100 Oe) of FeAl@Al_(1-x)_(Fe)_x_O_y_ and CoZn@Zn_(1-x)_Co_x_O_y_ NPs are plotted in Fig. 7. Their magnetic parameters are summarized in Table 2. The RTHLs of these nanohybrids (Fig. 7a, 7b) show the mono-phase nominal ferromagnetic feature with non-zero coercivities and small biases (~2 Oe). The reduced coercivities by comparing with those Co and Fe NPs less than 5 nm (Table 2)^43^ suggest that gradient doping ZnO or Al_2_O_3_ coatings on the Co or Fe cores can increase the magnetic dipole interaction among particles more greatly than the magnetic anisotropy, leading to the small magnetic reverse barrier, or reduced H_c_^9^,^17^,^38^,^43^. Clearly, the small exchange biases in these nanohybrids can be attributed to the exchange coupling between the cores and the surface metal oxide coatings by recalling their gradient core-shell structures. Their FC-ZFC curves show the highly bifurcated thermo-magnetism (Fig. 7c), or unmatched defreezing temperatures (T_f_) and merging temperature (T_m_), suggesting an enhanced magnetic anisotropy in these magnetic cores due to the gradient surface coatings. The higher T_f_ values for all nanohybrids than those of pure Co and Fe NPs indicate a strong pining interface indeed introduced between the cores and the surface coatings made of doped metal oxides. By considering the size and magnetic anisotropy effects on the T_f_ (T_f_ = *K_u_V*/25*k_B_*; *K_u_*: the magnetic anisotropy constant; *V*: the mean volume of the particles), the *K_u_* can be enhanced about 2.6 times for the FeAl@Al_(1-x)_Fe_x_O_y_ NPs (mean core diameter: 3 nm) compared with the Fe NPs. As for CoZn@Zn_(1-x)_Co_x_O_y_ NPs, the *K_u_* can be enhanced about 1.4 times for NPs (mean core diameter: 2.5 nm) synthesized by all NMP-phased reaction systems and about 1.7 times for NPs (core diameter: 2.5 nm) synthesized by aqueous-phased metal salt reaction systems. In addition, the exchange coupling effect between cores and shells on the *K_u_* cannot be excluded, which can reduce *K_u_*.

**Figure 8:**
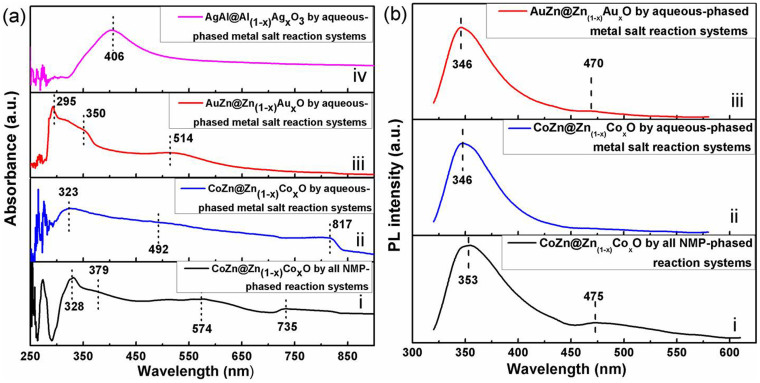
(a) UV-vis spectra of CoZn@Zn_(1-x)_Co_x_O_y_ (i) nanohybrids synthesized using using all NMP-phased reaction systems, and CoZn@Zn_(1-x)_Co_x_O_y_ (ii), AuZn@Zn_(1-x)_Au_x_O_y_ (iii) and AgAl@Al_(1-x)_Ag_x_O_y_ (iv) nanohybrids synthesized aqueous-phased metal salt reaction systems. (b) Photoluminescent spectra of CoZn@Zn_(1-x)_Co_x_O_y_ (i) synthesized using all NMP-phased reaction systems, and CoZn@Zn_(1-x)_Co_x_O_y_ (ii) and AuZn@Zn_(1-x)_Au_x_O_y_ (iii) nanohybrids synthesized using aqueous-phased metal salt reaction systems.

The optical properties of these nanohybrids having Zn_(1−x)_Co_x_O_y_, Ag, or Au components were examined by UV-vis (Fig. 8a) and photoluminescence spectroscopy (Fig. 8b). Generally, these nanohybrids with Zn_(1−x)_Co_x_O_y_ exhibit distinct blue shift UV absorbance and multi-peak feature as compared to that of bulk ZnO (~379 nm), which can be attributed to the bandgap shifts and different types of defects in ZnO caused by different doping and/or core effects. The CoZn@Zn_(1-x)_Co_x_O_y_ NPs synthesized using all NMP-phased reaction systems show multi-peak UV-vis absorbance (Fig. 8a-i): peaks at 328 nm and 379 nm are from ZnO, while peaks at 574 nm and 735 nm possibly are from defects caused by Co dopants. The CoZn@Zn_(1-x)_Co_x_O_y_ (Fig. 8a-ii) nanohybrids synthesized by aqueous-phased metal-salt reaction systems only show 2 peaks at 323 nm and 817 nm and one weak shoulder at 492 nm. This result suggests that more complicated doping effects on ZnO can be realized by using all NMP-phased reaction systems than by aqueous-phase metal salt reaction systems. The AgAl@Al_(1-x)_Ag_x_O_y_ (Fig. 8a-iv) and AuZn@Zn_(1-x)_Au_x_O_y_ NPs (Fig. 8a-iii) show distinct UV-vis absorbance at 406 nm and 514 nm, ascribed to the localized surface Plasmon resonance (LSPR) of Ag and Au cores affected by the thin Ag doping Al_2_O_3_ or Au doping ZnO shells, respectively. In addition, UV-vis absorbance of AuZn@Zn_(1-x)_Au_x_O_y_ NPs also shows two peaks at 294 nm and 350 nm, which may be the defect excitation in ZnO affected by the LSPR of Au cores.

The photoluminescence (PL) spectrum of the CoZn@Zn_(1-x)_Co_x_O_y_ nanohybrids synthesized using all NMP-phased reaction systems exhibits one distinct extinction PL peaks at 353 nm and one strong shoulder at 475 nm (Fig. 8b-i). The PL spectrum of these CoZn@Zn_(1-x)_Co_x_O_y_ nanohybrids synthesized using aqueous-phased metal-salt reaction systems gives blue shift ultra-violet extinction at ~346 nm and a suppressed blue extinction at ~475 nm (Fig. 8b-ii), possibly due to the reduced doping effect by considering their UV-vis spectra and reaction mechanisms. This can be further convinced by the much reduced peak at 470 nm for AuZn@Zn_(1-x)_Au_x_O_y_ NPs (Fig. 8b-iii) synthesized using aqueous-phased metal salt reaction systems since it may be more difficult for Au doping into ZnO shells than Co. Results on their UV-vis and PL spectra provide additional evidence for the formation of hybrid nanostructures with controlled surface coatings.

## Discussion

Generally, we have successful lly expanded SPMPs to a hybrid microfluidic and batch-cooling process for the synthesis of gradient core-shell nanohybrids based on the core alloying and shell gradient-doping strategy realized by coupled competitive reducing-nucleation and precipitation reactions. The crystal lattice mismatch between different components in nanohybrids can be efficiently avoided by utilizing this strategy. The coupled competitive reactions can be well-controlled by the types of mixed metal salts and bi-channel solvents in the microfluidics for the desired compositions and structures in metallic alloy cores and mixed metal oxide surface coatings. Most importantly, each stage in the nanoparticle formation can be spatiotemperally separated along the microfluidic channel and the rapid cooling receiver. Particularly, the sudden burst of transient nucleation to form alloy cores and the surface precipitation on preformed cores and the following growth stage can be efficiently separated from Ostwald ripening stage along the microfluidic channel. Thus, sizes, compositions and structures in alloy cores and surface coatings can be well-controlled at the desired growth stage. The crystal lattice mismatch between different components in nanohybrids can be efficiently avoided by utilizing the strategy of core alloying and gradient-doping the second metal oxide coating with the primary metal. Consequently, varieties of nanohybrids with metal alloys as cores and gradient-doping metal oxides as shells have been successfully synthesized, such as magneto-dielectrics (FeAl@Al_(1-x)_Fe_x_O_y_, CoAl@Al_(1-x)_Co_x_O_y_, CoFeAl@Al_(1-x)_(CoFe)_x_O_y_), magneto-semiconductors (FeZn@Zn_(1-x)_Fe_x_O_y_, CoZn@Zn_(1-x)_Co_x_O_y_), plasmon-semiconductors (AuZn@Zn_(1-x)_Au_x_O_y_) and plasmon-dielectrics (AgAl@Al_(1-x)_Ag_x_O_y_) (all x < 1). Results on nanohybrids except these in this article will be published in our future articles.

The coupled competitive reducing-nucleation and precipitation reactions can be performed either by all NMP-phased reaction systems or by aqueous-phased metal-salt reaction systems for a wide range of composition and structure control in these nanohybrids. The metal ratios in some nanohybrids measured by EDX were summarized in Table 3. Comparing with the theoretical values calculated by the metal content used in the feeds, ratios in these nanohybrids synthesized by all NMP-phased reaction systems are usually higher than the theoretical values, while it is on the contrary in those nanohybrids synthesized by aqueous-phased metal salt reaction systems. This result can be elucidated by the additional enhanced precipitation reactions of rx-14 and rx-15 besides rx1 ~ rx13^43^, leading to rapid formation of lots of Zn(OH)_2_ or Al (OH)_3_. These Al(OH)_3_ or Zn(OH)_2_ will precipitate on the cores to form relatively thick coatings, passivating the growth of metallic cores and finally leading to the reduced core contents (or the reduced primary metal content) in these nanohybrids. As Zn(OH)_2_ or Al(OH)_3_ are dehydrated to lose H_2_O, nanohybrids having Al_2_O_3_ or ZnO surface coatings will be formed and more Zn and Al contents can be introduced into metal oxide shells of nanohybrids than those formed in all NMP-phased reaction systems. Theoretically, this result may be related to their atomic ratios for binary alloy formation and the different precipitation rates under different reaction environments, which will be discussed in our future articles.
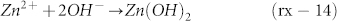

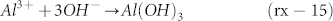


The primary metal to the second metal ratios in these nanohybrids calculated by XPS are also summarized in Table 3. As expected, these ratios are usually lower than the mean ratios in the whole nanohybrids measured by EDX since most surface coatings and only part of the metallic cores were included in the XPS measurement. This result provides additional evidence that the surface coatings are mainly made of primary metal doping second metal oxides and cores should be formed by the second metal alloying primary metal. The primary metal to the second metal ratio in CoZn@Zn_(1-x)_Co_x_O_y_ nanohybrids synthesized by aqueous-phased metal salt reaction systems is higher than those synthesized by all NMP-phased reaction systems even though the primary metal content used in all NMP-phased reaction systems is higher that that used in aqueous-phased metal salt reaction systems. This is consistent with the above analysis that aqueous-phased metal salt reaction systems favor to the rapid formation of Al(OH)_3_ or Zn(OH)_2_ that can precipitate on the cores to form relatively thick coatings.

These nanohybrids shall preserve unique magnetic and/or optical properties due to the gradient-doping in shells and core alloying that can significantly affect their surface structures and the proximity effects among components. The structure (sizes, surface coatings) and composition differences in nanohybrids originated from two reaction systems affect their magnetic properties significantly. According to the thermal magnetisms of CoZn@Zn_(1-x)_Co_x_O_y_ NPs (Table 2), the magnetic anisotropy in nanohybrids synthesized using aqueous-phased metal salt reaction systems is higher than those synthesized using all NMP-phased reaction systems. This result suggests that the rapid formation of surface oxides can greatly increase the surface pinning effect on magnetic cores and the interface canting effects between cores and surfaces. It is often evidenced by the chain-like nanoparticle assembles, as shown in Figure s6 using CoZn@Zn_(1-x)_Co_x_O_y_ nanohybrids as example. The thermal magnetism and relative K_u_ analyses of CoZn@Zn_(1-x)_Co_x_O_y_ and FeAl@Al_(1-x)_Fe_x_O_y_ NPs suggest that Al_2_O_3_ coatings can produce higher surface pining and interface canting effect than ZnO coatings. This result enlights us a promise for the synthesis of ultra-small ferromagnetic nanoparticles used at high temperature for ultra-high density media (Tbits/inch^2^) by greatly enhancing their magnetic anisotropies. Indeed, we have synthesize FeAl@Al_(1-x)_Fe_x_O_y_ and CoAl@Al_(1-x)_Co_x_O_y_ NPs of about 3.0 nm with T_f_ more than 300 K using aqueous-phased metal salt reaction systems, whose results will be published in our future articles.

Their UV-vis absorbances and photoluminescences indicate that the effective control of sizes and surface structures by suitable reaction routes in this article can conveniently tune their optical properties (Fig. 8). Generally, these nanohybrids with Zn_(1−x)_Co_x_O_y_ exhibit distinct blue shift UV absorbances compared with the bulk ZnO (~377 nm). Less types of defects can be introduced into Zn_(1−x)_Co_x_O_y_ by all NMP-phased reaction systems than by aqueous-phase reaction systems possibly due to the less Co dopant content in Zn_(1−x)_Co_x_O_y_ coatings. ZnO or Al_2_O_3_ coatings can influence their surface dielectric constants of AgAl@Al_(1-x)_Ag_x_O_y_ and AuZn@Zn_(1-x)_Au_x_O_y_ NPs (usually higher than water). UV-vis results on the newly synthesized AgAl@Al_(1-x)_Ag_x_O_y_ and Ag NPs (2.4 ± 0.2 nm, Fig. s7) suggest that the ultra-thin (~1 nm) Al_(1−x)_Ag_x_O_y_ coatings and Al alloying do not significantly affect the LSPR peak of such tiny Ag nanocrystals. However, UV-vis results on the newly synthesized AuZn@Zn_(1-x)_Au_x_O_y_ NPs and Au NPs (3.5 ± 0.3 nm, Fig. s7) suggest that the ultra-thin (<1 nm) Zn_(1−x)_Au_x_O_y_ coatings and Zn alloying can produce a blue shift of the LSPR peak in such tiny Au nanocrystals.

The core alloying and surface coating with different doping-metal types and contents also endow these nanohybrids with unique PLs. It is seen that all the spectra have a distinct peak of the intrinsic emission originated from the excitonic recombination in the UV region, corresponding to near-band-edge emission of zinc oxide, and/or a deep level emission peak in the visible region, corresponding to the intrinsic defect-related band. PL peaks in the UV region (346 ~ 353 nm) and in the blue region (470 nm or 475 nm) show blue-shift by comparing with the ZnO NPs (~376 nm and ~525 nm)^48^,^49^. The peak at the blue region (indicating the defect emission) for CoZn@Zn_(1-x)_Co_x_O_y_ nanohybrids synthesized by aqueous-phased metal salt reaction systems disappears. It can be reasonably explained by the greatly-reduced content of the primary metal in the metal oxide surface coating (XPS data in Table 3) synthesized using aqueous-phased metal-salt reaction systems since the deposition rates of ZnO on preformed cores can be significantly enhanced in the reaction system with high-water content (rx-14). However, the blue emission can still be observed in the nanohybrids synthesized using aqueous-phased reaction system if the core metal Co is replaced by Au. The combined results on PLs of nanohybrids synthesized using all NMP-phased reaction systems and aqueous-phased metal-salt reaction systems further enlight us that desired optical properties can be obtained by optimizing the composition and structure of cores and surface coatings. Indeed, greatly-enhanced blue emission were realized in FeZn@Zn_(1-x)_Co_x_O_y_ nanohybrids synthesized by all NMP-phased reaction systems, whose results will be published in our future articles.

These nanohybrids can be concentrated into slurry by centrifugal or magnets and dried to powder, which can be re-dispersed in high-polar solvents (e.g., NMP or H_2_O or chlorobenzene, Fig. s8a–c) and/or low-polar solvents (e.g., chloroform or hexane, Fig. s8d–e) and show different colors. Their good solubilities in either polar or non-polar solvents (Table 4) endow them with broader applications than those NPs dissolved solely in polar or non-polar solvents. Applications on these kinds of NPs and other kinds of nanohybrids (FeZn@Zn_(1-x)_Co_x_O_y_, CoAl@Al_(1-x)_Co_x_O_y_, NiPt@Ni_(1-x)_Pt_x_O_y_, AgRu@Ru_(1-x)_Ag_x_O_2_, AuRu@Ru_(1-x)_Au_x_O_y_, FeMn@Mn_(1-x)_Fe_x_O_y_, FePt@FeOOH, etc.) synthesized by this strategy have been evaluated either as dual contrast agents for magnetic resonance imaging, or as energy storage materials for supercapacitors, or as optical emitters for optoelectronic devices, or as efficient catalysts for fuel cell, which will be discussed in our future articles. All these amazing applications and their unique physicochemical properties are, no doubt, due to the core alloying and shell gradient-doping in these nanohybrids. We believe that the developed sequenced reducing-nucleation and precipitation reaction in microfluidics shall provide us a general low cost scale out approach in the composition and structure controlled synthesis of nanohybrids with defined properties for advanced applications.

## Experiment Methods

### Assembly of the mixed microfluidic and batch-cooling process (hybrid process)

The hybrid process was designed and assembled by connecting preheating stainless steel tubing coils, a transparent polymer tubing (e.g., Teflon) a Y connector (e.g. PEEK), the thermostatic tank 1 and 2 and the cooling tank for product receivers, as shown in Fig. s1.

### Sequenced in-situ redox of metal ions and/or precipitation of metal oxides for hybrid nanoparticle synthesis

This synthesis method was developed by directly introducing the second-metal-salt with low standard electrode potential (i.e., Zn^2+^: V_0_ = −0.763 V; Al^3+^: V_0_ = −1.662 V) into the primary-metal-salt solutions in the hybrid process. The detailed synthesis conditions for these hybrid NPs were summarized in Table 1 and Part I of SI.

### Composition and structure characterization of nanoparticles

Morphologies, chemical compositions and lattice fringes of the nanowires were characterized by transmission electron microscopy (TEM; JOEL 2100F, 200 kV) equipped with energy-dispersive X-ray spectroscopy (EDX). The STEM-HAADF images (Z-contrast image) were obtained on a Tecnai F20 (200 kV) electron microscope equipped with EDX for point-by-point element analysis in single nanoparticles. The X-ray photoelectron spectroscopy (XPS) was used to determine the elemental composition as well as chemical and electronic state of the related elements in NPs by detecting their thin films. XPS measurements were carried out on an ESCALAB 250 Thermo Electron Corporation with an Al Kα X-ray source (1486.6 eV photons). The core-level signals were obtained at a photoelectron take-off angle of 45° (with respect to the sample surface). The X-ray source was run at a power of 300 W. The samples were mounted on the standard sample studs by means of double-sided adhesive tapes. The pressure in the analysis chamber was maintained at 2 × 10^−9^ mbar during each measurement. To compensate for surface charging effects, all binding energies (BE's) were referenced to the C 1s hydrocarbon peak at 284.6 eV. Powder X-ray diffraction (XRD) data of samples were collected on a D/max 2200PC diffractometer (Cu Kα radiation, λ = 1.5418 Å, Rigaku, Japan).

### Characterization of magnetic and optical property

The magnetic properties were evaluated by room temperature hysteresis loop (RMHL) and/or thermo-magnetism (ZFC: zero-field cooling; FC: field cooling) curves measured by MPPS (SQUID) (Quantum design) using an applied magnetic field of 100 Oe. The UV-vis spectra of samples in different solvents were measured by UV-vis spectroscopy (TU-1901, 190–900 nm, Beijing Purkinje General Instrument Co., Ltd.) using the corresponding solvent as a reference. The photoluminescence (PL) property of samples was performed at room temperature by using 310 line of Xe 900 as the excitation source (FL-920, 450 W, Shanghai Qianxin Instrument Inc.).

## Author Contributions

Yujun S. conceived the idea and designed the project, discussed with W.Y. and R.W. Yujun S., X.S., J.W., S.L., Yuan.S., X.Z. and R.W. carried out the nanoparticles synthesis, XRD measurements, TEM, HR-TEM, UV-vis, PL and EDX characterization. W.Y., Yujun S., X.S. and J.W. did the XPS and magnetic measurement. R.W., Yuan.S. and Yujun S. conducted the STEM-HAADF characterization. Yujun S., R.W., W.Y., Yuan.S. and J.W. did the data analysis. Yujun S., W.Y. and R.W. wrote the paper.

## Supplementary Material

Supplementary Information Supplementary information

## Figures and Tables

**Table 1:** Synthesis conditions of nanohybrids

Methods	Nanoparticles	Size nm	Metal salts mM (mmol/L)	NaBH_4_ mM	Stabilizers mM	Reaction Temperature (T) °C	Receiver T °C	Flow rate mL/min
All NMP-phased reaction systems	FeAl@Al_(1-x)_Fe_x_O_y_	4.8 ± 0.3	FeCl_2_·4H_2_O: 27; AlCl_3_: 12	120	PVP: 0.94	80	10	1.0
	CoZn@Zn_(1-x)_Co_x_O_y_	3.5 ± 0.3	CoCl_2_·6H_2_O: 32; ZnCl_2_: 120	1016	PVP: 0.84	80	10	0.8
Aqueous-phased metal-salt reaction systems	CoZn@Zn_(1-x)_Co_x_O_y_	4.4 ± 0.4	CoCl_2_·6H_2_O: 10; ZnCl_2_: 3	175	PVP: 0.7; VC: 5.5	80	10	3.0
	AuZn@Zn_(1-x)_Au_x_O_y_	3.6 ± 0.2	HAuCl_4_·xH_2_O: 29; ZnCl_2_: 50	180	PVP: 0.92	80	10	3.0
	AgAl@Al_(1-x)_Ag_x_O_y_	3.7 ± 0.3	AgNO_3_: 25; AlCl_3_: 10	175	PVP: 0.46	80	10	5.0

PVP: polyvinylpyrrolidone; VC: vitamin C.

**Table 2:** Magnetic properties of nanohybrids

			a H_c_, Oe		b T_f_ K	c T_m_ K
Method	Sample	Size nm	left	right		
All NMP phased reaction systems	FeAl@Al_(1-x)_Fe_x_O_y_	4.8 ± 0.3	−11	9	235	295
	CoZn@Zn_(1-x)_Co_x_O_y_	3.5 ± 0.3	−27	25	109	398
Aqueous phased metal salt reaction systems	CoZn@Zn_(1-x)_Co_x_O_y_	4.4 ± 0.4	−11	9	132	398
All NMP phased reaction systems	d Fe	3.5 ± 0.3	−37	37	141	348
	d Co	2.5 ± 0.2	−35	34	73	146

^a^Hc: coercivity;

^b^T_f_: the freezing temperature above which the NPs gradually shift from clusters-glass (CG) like state to the ferromagnetic (FM) state;

^c^T_m_: the merging temperature for zero-field-cooling curves (M_ZFC_(T)) and field-cooling curves (M_FC_(T)), indicating that the NPs are in the same FM state for ZFC and FC processes above that temperature;

^d^Data from X Shen, et al., RSC Advances 4, 34179(2014).

**Table 3:** Theoretical (feeding content) and experimental (by EDX) metal atom ratios of nanohybrids and surface metal atom ratios in nanohybrids by XPS

Method	Sample	Size, nm	Theoretical atomic ratio	Atomic ratio of whole particles by EDX	Top surface atomic ratio by XPS
All NMP-phased reaction systems	CoZn@Zn_(1-x)_Co_x_O_y_	3.5 ± 0.3	Co/Zn: 21/79	Co/Zn: 43/57	Co/Zn: 31/69
	FeAl@Al_(1-x)_Fe_x_O_y_	4.8 ± 0.3	Fe/Al: 76/24	Fe/Al: 82/18	Fe/Al: 34/66
Aqueous-phased metal salt reaction systems	CoZn@Zn_(1-x)_Co_x_O_y_	4.4 ± 0.4	Co/Zn: 77/23	Co/Zn: 23/77	Co/Zn: 20/80
	AgAl@Al_(1-x)_Ag_x_O_y_	3.7 ± 0.3	Ag/Al: 71/29	Ag/Al: 38/62	Ag/Al: 5/95
	AuZn@Zn_(1-x)_Au_x_O_y_	3.6 ± 0.2	Au/Zn: 37/64	Au/Zn: 18/82	Au/Zn: 5/95

**Table 4:** Solubility of nanohybrids in different solvents by directly re-dispersing their dry powder

		Solubility in solvents				
		H_2_O	NMP	Chlorobenzene	Chloroform	Cyclohexane
No	Nanoparticles	(a)	(b)	(c)	(d)	(e)
1	* CoZn@Zn_(1-x)_Co_x_O_y_	>10 g/L	5–10 g/L	>10 g/L	2–5 g/L	5–10 g/L
2	* FeAl@Al_(1-x)_Fe_x_O_y_	>10 g/L	>10 g/L	2–5 g/L	2–5 g/L	5–10 g/L
3	# CoZn@Zn_(1-x)_Co_x_O_y_	>10 g/L	>10 g/L	2–5 g/L	5–10 g/L	5–10 g/L
4	# AuZn@Zn_(1-x)_Au_x_O_y_	>10 g/L	>10 g/L	5–10 g/L	>10 g/L	>10 g/L
5	# AgAl@Al_(1-x)_Ag_x_O_y_	>10 g/L	>10 g/L	>10 g/L	>10 g/L	>10 g/L

*synthesized by all NMP-phased reaction systems.

#synthesized by aqueous-phased metal-salt reaction systems.

## References

[b1] Wu B., Kuang Y., Zhang X. & Chen J. Noble metal nanoparticles/carbon nanotubes nanohybrids: Synthesis and applications. Nano Today 6, 75–90 (2011).

[b2] Huang J. *et al.* Crystal engineering and SERS properties of Ag–Fe3O4 nanohybrids: from heterodimer to core–shell nanostructures. J. Mater. Chem. 21, 17930–17937 (2011).

[b3] Song Y., Wang Y., Li B. B., Fernandes C. & Ruda H. E. Interfacial Interaction Induced Self-Assembly of Nanoparticles into Superstructures. Nanoscale 5, 6779–6789 (2013). 23793729 10.1039/c3nr01366a

[b4] Song Y. *et al.* Magneto-Plasmons in Periodic Nanopore Structures. Sci. Rep. 4, 4991 (2014).

[b5] Belotelov V. I. *et al.* Plasmon-mediated magneto-optical transparency. Nat. Commun. 4, 2128 (2013). 23839481 10.1038/ncomms3128 PMC3717503

[b6] Belotelov V. I. *et al.* Enhanced magneto-optical effects in magnetoplasmonic crystals. Nat. Nanotechnol. 6, 370–376 (2011). 21516090 10.1038/nnano.2011.54

[b7] Montero M. I. *et al.* Nanostructures and the proximity effect. J. Phys. D: Appl. Phys. 35, 2398–2402 (2002).

[b8] Song Y. *et al.* Magnetic and electric property evolution of amorphous cobalt-rich alloys driven by field annealing. J. Phys. D: Appl. Phys. 45, 225001 (2012).

[b9] Song Y., Ding J. & Wang Y. Shell Dependent Evolution of Optical and Magnetic Properties of Co@Au Core-shell Nanoparticles. J. Phys. Chem. C 116, 11343–11350 (2012).

[b10] Cozzoli P. D., Pellegrino T. & Manna L. Synthesis, properties and perspectives of hybrid nanocrystal structures. Chem. Soc. Rev. 35, 1195–1208 (2006). 17057845 10.1039/b517790c

[b11] Song Y., Wang Y. H., JI S. & Ding J. Shell-Driven Fine Structure Transition in Co@Au Core-shell Nanoparticles. Nano-Micro Lett. 4, 235–242 (2012).

[b12] Sun Q., Ren Z., Wang R., Wang N. & Cao X. Platinum Catalyzed Growth of NiPt Hollow Spheres with an Ultrathin Shell. J. Mater. Chem. 21, 1925–1930 (2011).

[b13] Luo J. *et al.* Homogeneous Photosensitization of Complex TiO2 Nanostructures for Efficient Solar Energy Conversion. Sci. Rep. 2, 451 (2012). 22693653 10.1038/srep00451 PMC3372876

[b14] Song Y., Sun P., Henry L. L. & Sun B. Mechanism for Structure and Performance Controlled Preparation of Thin Film Composite Membrane via Interfacial Polymerization. J. Membr. Sci. 251, 67–72 (2005).

[b15] Wang L. *et al.* Hierarchical SnO_2_ Nanospheres: Bio-inspired Mineralization, Vulcanization, Oxidation Techniques, and the Application for NO Sensors. Sci. Rep. 3, 3500 (2013). 24336171 10.1038/srep03500 PMC3863821

[b16] Li S. *et al.* Controlled Hybridization of Sn-SnO2 Nanoparticles via Simple Programmed Microfluidic Processes for Tunable Ultraviolet and Blue Emissions. J. Mater. Chem. C 2, 7687–7694 (2014).

[b17] Song Y. *et al.* In-situ Redox Microfluidic Synthesis of Core-Shell Nanoparticles and their Long-term Stability. J. Phys. Chem. C 117, 17274–17284 (2013).

[b18] Temnov V. V. The marriage of plasmonics, magnetism, acoustics and ultrafast optics. Nat. Photonics 6, 728–736 (2012).

[b19] Jin Y., Jia C., Huang S.-W., O'Donnell M. & Gao X. Multifunctional nanoparticles as coupled contrast agents. Nat. Commun. 1, 41 (2010). 20975706 10.1038/ncomms1042 PMC3205955

[b20] Murphy C. J. *et al.* Anisotropic Metal Nanoparticles: Synthesis, Assembly, and Optical Applications. J. Phys. Chem. B 109, 13857–13870 (2005). 16852739 10.1021/jp0516846

[b21] Burda C., Chen X., Narayanan R. & El-Sayed M. A. Chemistry and Properties of Nanocrystals of Different Shapes. Chem. Rev. 105, 1025–1102 (2005). 15826010 10.1021/cr030063a

[b22] Bach L. G., Islam M. R., Lee D. C. & Lim K. T. Poly(glycidyl methacrylate) grafted CdSe quantum dots by surface-initiated atom transfer radical polymerization: Novel synthesis, characterization, properties, and cytotoxicity studies. Appl. Surf. Sci. 283, 546–553 (2013).

[b23] Chen G. *et al.* Interfacial Effects in Iron-Nickel Hydroxide–Platinum Nanoparticles Enhance Catalytic Oxidation. Science 344, 495–499 (2014). 24786074 10.1126/science.1252553

[b24] Nan F. *et al.* Manipulating Nonlinear Emission and Cooperative Effect of CdSe/ZnS Quantum Dots by Coupling to a Silver Nanorod Complex Cavity. Sci. Rep. 4, 4839 (2014). 24787617 10.1038/srep04839 PMC4007083

[b25] Farmer J. A. & Campbell C. T. Ceria Maintains Smaller Metal Catalyst Particles by Strong Metal-Support Bonding. Science 329, 933–935 (2010). 20724631 10.1126/science.1191778

[b26] deMello A. J. Control and detection of chemical reactions in microfluidic systems. Nature 442, 394–402 (2006). 16871207 10.1038/nature05062

[b27] Song Y., Kumar C. S. S. R. & Hormes J. Microfluidic Synthesis of Nanomaterials. Small 4, 698–711 (2008). 18535993 10.1002/smll.200701029

[b28] Marre S. & Jensen K. F. Synthesis of micro and nanostructures in microfluidic systems. Chem. Soc. Rev. 39, 1183–1202 (2010). 20179831 10.1039/b821324k

[b29] Nightingale A. M. *et al.* Large-scale synthesis of nanocrystals in a multichannel droplet reactor. J. Mater. Chem. A 1, 4067–4076 (2013).

[b30] Song Y. & Henry L. L. Nearly Monodispersion CoSm Nanoparticles Synthesized by a Microfluidic reactor. Nanoscale Res. Lett. 4, 1130–1134 (2009). 20596324 10.1007/s11671-009-9369-8 PMC2894255

[b31] Carroll K. J. *et al.* One-Pot Aqueous Synthesis of Fe and Ag Core/Shell Nanoparticles. Chem. Mater. 22, 6291–6296 (2010).

[b32] Boross P. T., Dóra B. Z., Kiss A. R. & Simon F. A unified theory of spin-relaxation due to spin-orbit coupling in metals and semiconductors. Sci. Rep. 3, 3233 (2013). 24252975 10.1038/srep03233 PMC3834866

[b33] Zhang J., Tang Y., Lee K. & Ouyang M. Nonepitaxial growth of hybrid core-shell nanostructures with large lattice mismatches. Science 327, 1634–1638 (2010). 20339071 10.1126/science.1184769

[b34] Seo W. S. *et al.* FeCo/graphitic-shell nanocrystals as advanced magnetic-resonance-imaging and near-infrared agents. Nat. Mater. 5, 971–976 (2006). 17115025 10.1038/nmat1775

[b35] Wang X., Zhuang J., Peng Q. & Yadong L. A general strategy for nanocrystals synthesis. Nature 437, 121–124 (2005). 16136139 10.1038/nature03968

[b36] Khan S. A. & Duraiswamy S. Controlling bubbles using bubbles-microfluidic synthesis of ultra-small gold nanocrystals with gas-evolving reducing agents. Lab Chip 12, 1807–1812 (2012). 22456754 10.1039/c2lc21198j

[b37] Song Y. *et al.* Investigations into sulfobetaine-stabilized Cu nanoparticle formation: toward development of a microfluidic synthesis. J. Phys. Chem. B 109, 9330–9338 (2005). 16852117 10.1021/jp044777g

[b38] Song Y., Henry L. L. & Yang W. T. Stable Cobalt Amorphous Nanoparticles Formed by an In-situ Rapid Cooling Microfluidic Process. Langmuir 25, 10209–10217 (2009). 19601562 10.1021/la9009866

[b39] Abou-Hassan A., Bazzi R. & Cabuil V. Multistep Continuous-Flow Microsynthesis of Magnetic and Fluorescent g-Fe_2_O_3_@SiO_2_ Core/Shell Nanoparticles. Angew. Chem. Int. Ed. 48, 7180–7183 (2009). 10.1002/anie.200902181 19708045

[b40] Park J. I., Saffari A., Kumar S., Gunther A. & Kumacheva E. Microfluidic synthesis of polymer and inorganic particulate materials. Annu. Rev. Mater. Res. 40, 415–443 (2010).

[b41] Erdem E. Y., Cheng J. C., Doyle F. M. & Pisano A. P. Multi-Temperature Zone, Droplet-based Microreactor for Increased Temperature Control in Nanoparticle Synthesis. Small 10, 1076–1080 (2014). 24339202 10.1002/smll.201302379

[b42] Song Y., Kumar C. S. S. R. & Hormes J. Fabrication of SU-8 based microfluidic reactor on a PEEK substrate sealed by a “flexible semi-solid transfer” (FST) process. J. Micromech. Microeng. 14, 932–940 (2004).

[b43] Shen X. *et al.* Spatiotemporal-Resolved Ultra-small Nanoparticle Synthesis via Simple Programmed Microfluidic Processes. RSC Adv. 4, 34179–34188 (2014).

[b44] James B. D. & Wallbride M. G. H. Metal Tetrahydroborate. Vol. 11, 99–232 (Interscience Publisher, 1970).

[b45] Mochalov K. N. & Tremasov N. V. Nature of the Products and Mechanism of the Reaction of BH4- with Heavy Metals. Vol. 36, 48–55 (Khim-Tekhn. Inst., 1967).

[b46] Kollonitsch J. & Fuchs O. Preparation of Aluminium borohydride and its application in organic reductions. Nature 176, 1081–1081 (1955).

[b47] Jeon E. & Cho Y. Mechanochemical synthesis and thermal decomposition of zinc borohydride. J. Alloy Comp. 422, 273–275 (2006).

[b48] Pal B. & Giri P. K. Room Temperature Ferromagnetism in Co-Doped ZnO Nanoparticles: Milling Time Dependence and Annealing Effect. Int. J. Nanosci. 10, 1–5 (2011).

[b49] Song J.-L., Zheng J.-H., Zhao Z., Zhou B.-Y. & Lian J.-S. Synthesis and photoluminescence of Y and Cd co-doped ZnO nanopowder. Trans. Nonferrous Met. Soc. China 23, 2336–2340 (2013).

