# Supplementary material for: A General Strategy for Nanohybrids Synthesis via Coupled Competitive Reactions Controlled in a Hybrid Process
Source: Sci Rep. 2015 Mar 30;5:9189. doi: 10.1038/srep09189 (PMC4377631; doi:10.1038/srep09189)
Supplement: Supplementary Information [file srep09189-s1.pdf]

Supporting Information for

A General Strategy for Nanohybrids Synthesis via Coupled Competitive Reactions Controlled in a Hybrid Process

Rongming Wang[1#], Wantai Yang[2#], Yuanjun Song[1], Xiaomiao Shen[3], Junmei Wang[1], Xiaodi Zhong[3], Shuai Li[3], Yujun Song[1]*

[1] Department of Physics, School of Mathematics and Physics, University of Science & Technology Beijing, Beijing 100083, China; [2] College of Materials and Engineering, Beijing University of Chemical Technology, Beijing 100029, China; [3]School of Materials Science and Engineering, Beihang University, Beijing 100191, China

* Corresponding authors: Yujun S., songyj@ustb.edu.cn

[#] Contribution to this article equally.

Part I: Detailed synthesis conditions of nanoparticles

(1) $FeAl@Al_{(1-x)}Fe_xO_y$ hybrid NPs with different solution color formed at different reaction stage using all NMP-phased reaction systems

Reactant solution preparation. PVP of 0.468 g (Mw = 10000), $AlCl_3$ of 0.0683 g (0.50 mmol) and $FeCl_2{\cdot}4H_2O$ of 0.320 g (1.61 mmol) are dissolved into 50mL NMP to form the metal salt solution. $NaBH_4$ of 0.341 g (9.0 mmol) is dissolved into 50 mL NMP to form the reducing solution.

Reaction conditions. The microfluidic synthesis is performed at 80°C under inert atmosphere (nitrogen) protection using the following procedure: 20 mL of metal salt solution and 20 mL of reducing solution are sucked into each of the syringes and fixed in the platform of the syringe pump, which is introduced into the Y mixer (e) to start the reaction after preheating by the stainless steel coils in tank 1 at a flow rate of 1.0 mL/min per pump and then the solution enters into the microchannel (f) to finish the growth of NPs.

Sample collection for the reaction solution color observation and TEM sample preparation at each stage. Samples for each stage were obtained by tuning the residence time in microchannel, which is controlled by the microchannel length and diameter (here; 250 μm) in the tank 2. Then the reaction solution was collected in a sample bottle cooled in the tank 3 with temperature of about 2°C. The microchannel lengths for the sample of the white intermediate solution (Fig. 1a), for the light-brown sample that the nucleation and short growth just occur (Fig. 1b), for the brown sample that a little bit long growth and an initial Ostwald ripening occur (Fig. 1c) and for the sample that a little bit long Ostwald ripening and an initial aggregation occurs are controlled at 10 cm, 15 cm, 35 cm and 60 cm, respectively. The calculated residence time (t_r) for each sample in the microchannel is 0.39 s, 0.44 s, 1.03 s and 1.77 s, respectively by the equation of $t_r = \pi d^2 L_{total}/(4Q)$ (d: inner diameter of the microchannel; L_{total}: channel length for the reaction; Q: the flow rate controlled by the syringe pump and calibrated by the real flow rate in experiments). The sample bottle was taken from the cooling tank 3, and placed on the table quickly, and then a color picture of the solution was taken as fast as possible. The sample for TEM observation was prepared by dropping the cooled sample solution on the TEM grid, and the solution on the grid was absorbed quickly by a sharp tip filter paper, and then the grid was dried under vacuum as quickly as possible at room temperature.

(2) Synthesis of $FeAl@Al_{(1-x)}Fe_xO_y$ hybrid NPs using all NMP-phased reaction systems

Briefly, PVP of 0.468 g (Mw = 10000), $AlCl_3$ of 0.0683 g (0.50 mmol) and $FeCl_2{\cdot}4H_2O$ of 0.320 g (1.61 mmol) are dissolved into 50mL NMP to form the metal salt solution. $NaBH_4$ of 0.341 g (9.0 mmol) is dissolved into 50 mL NMP to form the reducing solution. Then the microfluidic synthesis is performed at 80°C under inert atmosphere (nitrogen) protection using the following procedure: 20 mL of metal salt solution and 20 mL of reducing solution are sucked into each of the syringes and fixed in the platform of syringe pump, which is introduced into the Y mixer (e) to finish the nucleation by the syringe pump at a flow rate of 1.0 mL/min per pump and then the solution enters into the microchannel (f) to finish the growth of NPs. The obtained fresh NP solution is collected in the product receiver (g) cooled

by the thermostatic tank 3. The NPs are precipitated using centrifuge at a speed of 15000 rpm for 20min and the top supernatant is decanted. The precipitated NPs are dissolved into the same volume of NMP. The centrifuge process is repeated twice and the final black slurry in the bottle is dried under vacuum and kept in the desiccators for future use. Part of the sample is stored in the air or re-dissolved into DI water for the long-term stability evaluation.

(3) Synthesis of $CoZn@Zn_{(1-x)}Co_xO_y$ hybrid NPs using all NMP-phased reaction systems

Briefly, PVP of 0.42 g (Mw = 10000), $ZnCl_2$ of 0.818 g (6.0 mmol) and $CoCl_2{\cdot}6H_2O$ of 0.386 g (1.6 mmol) are dissolved into 50 mL NMP to form the metal salt solution. $NaBH_4$ of 1.92 g (50.8 mmol) is dissolved into 50 mL NMP to form the reducing solution. Then the microfluidic synthesis is performed at 78°C under inert atmosphere (nitrogen) protection using the following procedure: 20 mL of metal salt solution and 20 mL of reducing solution are sucked into each of the syringes and fixed in the platform of syringe pump, which is introduced into the Y mixer (e) to finish the nucleation by the syringe pump at a flow rate of 0.5 mL/min per pump and then the solution enters into the microchannel (f) to finish the growth of NPs. The obtained fresh NP solution is collected in the product collector (g). The NPs are precipitated using centrifuge at a speed of 15000 rpm for 20min and the top supernatant is decanted. The precipitated NPs are dissolved into the same volume of NMP. The centrifuge process is repeated twice and the final black slurry in the bottle is dried under vacuum and kept in the desiccators for future use. Part of the sample is stored in the air or re-dissolved into DI water for the long-term stability evaluation.

(4) Synthesis of $CoZn@Zn_{(1-x)}Co_xO_y$ hybrid NPs using aqueous-phased metal salt reaction systems.

Briefly, PVP of 0.35 g (Mw = 10000), 0.119 g (0.5 mmol) of $CoCl_2{\cdot}6H_2O$and 0.170 g (0.125 mmol) of ZnCl2 are dissolved into 50 mL ultrapure water to form the metal salt solution. NaBH4 of 0.331 g (8.75 mmol) is dissolved into 50 mL NMP to form the reducing solution. Then the microfluidic synthesis is performed at 20°C using the following procedure: 20 mL of metal salt solution and 20 mL of reducing solution are sucked into each of the syringes and fixed in the platform of syringe pump, which is introduced into the Y mixer (e) to finish the nucleation by the syringe pump at a flow rate of 3.0 mL/min per pump and then the solution enters into the microchannel (f) to finish the growth of NPs. The obtained fresh NP solution is collected in the product collector (g). The NPs are precipitated using centrifuge at a speed of 15000 rpm for 20min and the top supernatant is decanted. The precipitated NPs are dissolved into the same volume of ultrapure water. The centrifuge process is repeated twice and the final slurry in the bottle is dried under vacuum and kept in the desiccators for future use. Part of the sample is stored in the air or re-dissolved into DI water for the long-term stability evaluation.

(5) Synthesis of $AgAl@Al_{(1-x)}Ag_xO_y$ hybrid NPs using aqueous phased metal salts using aqueous-phased metal salt reaction systems.

Briefly, PVP of 0.23 g (Mw = 10000), 0.212 g (1.25 mmol) of $AgNO_3$ and 0.068 g (0.05 mmol) of $AlCl_3$ are dissolved into 50 mL ultrapure water to form the metal salt solution. NaBH4 of 0.331 g (8.75 mmol) is dissolved into 50 mL NMP to form the reducing solution.

Then the microfluidic synthesis is performed at 20°C using the following procedure: 20 mL of metal salt solution and 20 mL of reducing solution are sucked into each of the syringes and fixed in the platform of syringe pump, which is introduced into the Y mixer (e) to finish the nucleation by the syringe pump at a flow rate of 5.0 mL/min per pump and then the solution enters into the microchannel (f) to finish the growth of NPs. The obtained fresh NP solution is collected in the product collector (g). The NPs are precipitated using centrifuge at a speed of 15000 rpm for 20min and the top supernatant is decanted. The precipitated NPs are dissolved into the same volume of ultrapure water. The centrifuge process is repeated twice and the final slurry in the bottle is dried under vacuum and kept in the desiccators for future use. Part of the sample is stored in the air or re-dissolved into DI water for the long-term stability evaluation.

(6) Synthesis of AuZn@$Zn_{(1-x)}Au_xO_y$ alloy hybrid NPs using aqueous phased metal salts using aqueous-phased metal salt reaction systems.

Briefly, PVP of 0.46 g (Mw = 10000), $ZnCl_2$ of 0.339 g (2.49 mmol) and $HAuCl_4$ of 0.5 g (1.47 mmol) are dissolved into 50mL ultrapure water to form the metal salt solution. $NaBH_4$ of 0.34 g (8.98 mmol) is dissolved into 50 mL NMP to form the reducing solution. Then the microfluidic synthesis is performed at 80°C under inert atmosphere (nitrogen) protection using the following procedure: 20 mL of metal salt solution and 20 mL of reducing solution are sucked into each of the syringes and fixed in the platform of syringe pump, which is introduced into the Y mixer (e) to finish the nucleation by the syringe pump at a flow rate of 3.0 mL/min per pump and then the solution enters into the microchannel (f) to finish the growth of NPs. The obtained fresh NP solution is collected in the product collector (g). The NPs are precipitated using centrifuge at a speed of 15000 rpm for 20min and the top supernatant is decanted. The precipitated NPs are dissolved into the same volume of NMP. The centrifuge process is repeated twice and the final black slurry in the bottle is dried under vacuum and kept in the desiccators for future use. Part of the sample is stored in the air or re-dissolved into DI water for the long-term stability evaluation.

(7) Synthesis of Au NPs

Briefly, PVP of 0.4 g (Mw = 10000), maleic aldehyde (MAH) of 0.4 g (4.06 mmol) and tri-sodium citrate (TSC) of 1.77 g (6.02 mmol) are dissolved into 50 mL ultrapure water to form the reducing solution and HAuCl4 of 0.11 g is dissolved into 50 mL NMP to form the metal salt solution. Then the microfluidic synthesis is performed at 90°C using the following procedure: 20 mL of metal salt solution and 20 mL of reducing solution are sucked into each of the syringes and fixed in the platform of syringe pump, which is introduced into the Y mixer (e) to finish the nucleation by the syringe pump at a flow rate of 7.0 mL/min per pump and then the solution enters into the microchannel (f) to finish the growth of NPs. The obtained fresh NP solution is collected in the product collector (g) at 2^oC. The NPs are precipitated using centrifuge at a speed of 20000 rpm for 60 min and the top supernatant is decanted. The precipitated NPs are dissolved into the same volume of ultrapure water. The centrifuge process is repeated several times and all the slurry in the bottle for each time are dried under vacuum and kept in the desiccators for future use. Part of the slurry is stored in the air or re-dissolved into DI water for the long-term stability evaluation.

(8) Synthesis of Ag NPs

Briefly, PVP of 0.4 g (Mw = 10000), TSC of 0.3 g (Mw = 258.07) and $N_2H_4 \cdot H_2O$ of 0.022 ml (~1.10 mmol) are dissolved into 50 mL ultrapure water to form the reducing solution $AgNO_3$ of 0.25 g (1.47 mmol) is dissolved into 50 mL NMP to form the metal salt solution. Then the microfluidic synthesis is performed at 80°C using the following procedure: 20 mL of metal salt solution and 20 mL of reducing solution are sucked into each of the syringes and fixed in the platform of syringe pump, which is introduced into the Y mixer (e) to finish the nucleation by the syringe pump at a flow rate of 5.0 mL/min per pump and then the solution enters into the microchannel (f) to finish the growth of NPs. The obtained fresh NP solution is collected in the product collector (g) at 2^oC. The NPs are precipitated using centrifuge at a speed of 20000 rpm for 60 min and the top supernatant is decanted. The precipitated NPs are dissolved into the same volume of ultrapure water. The centrifuge process is repeated several times and all the slurry in the bottle for each time are dried under vacuum and kept in the desiccators for future use. Part of the slurry is stored in the air or re-dissolved into DI water for the long-term stability evaluation.

Part II: Figure s1-s8

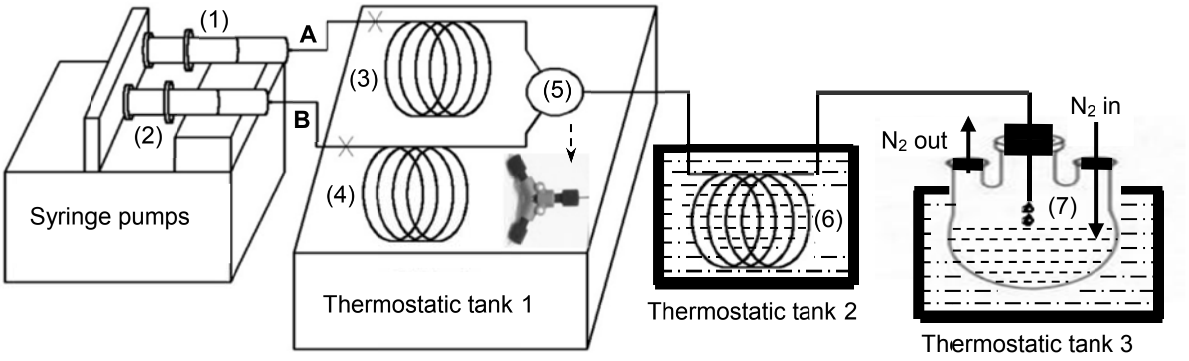

Figure s1 Experiment setup of micro-tubing based simple programmed microfluidic processes (SPMPs)：(1) and (2), syringe pumps for reducing agent solution and metal salt solution; (3) and (4), microtubing coils for pre-heating reducing agent solution and metal salt solution with the temperature controlled by thermostatic tank 1; (5), three-way mixer for reaction between reducing agent solution and metal salt solution; (6), microtubing coil for nucleation and nanoparticle growth with temperature controlled by thermostatic tank 2; (7), nanoparticle collector with temperature controlled by thermostatic tank 3, where the growth is terminated at a designed temperature. In all stages, the temperature can be currently controlled from -15°C to 200°C based on the melting and boiling points of the solvent NMP (T_m: -24°C; T_b: 202°C). The pressure drop in the microchannel depends on the microchannel length and the flow rate controlled the syringe pump and calculated by the pressure in capillary as below.[1,2]

$$\Delta P = Q\frac{C_{fr}L\mu}{2AD_h^2}$$

Where, C_{fr} = 64 is the friction coefficient for circular cross section; A is the cross section area of the flow; D_h is the hydraulic diameter; μ is the fluid dynamic viscosity and L is the channel length. The calculated pressure drops for microchannel with diameter of 250 μm is about 0.23MPa for L = 10 cm, 0.35 MPa for L = 15 cm and 1.39MPa for L = 60 cm if using the fluid dynamic viscosity of NMP at 25°C (13.3 mPa·s). Clearly, the pressure drop at 80°C should be lower than these values.

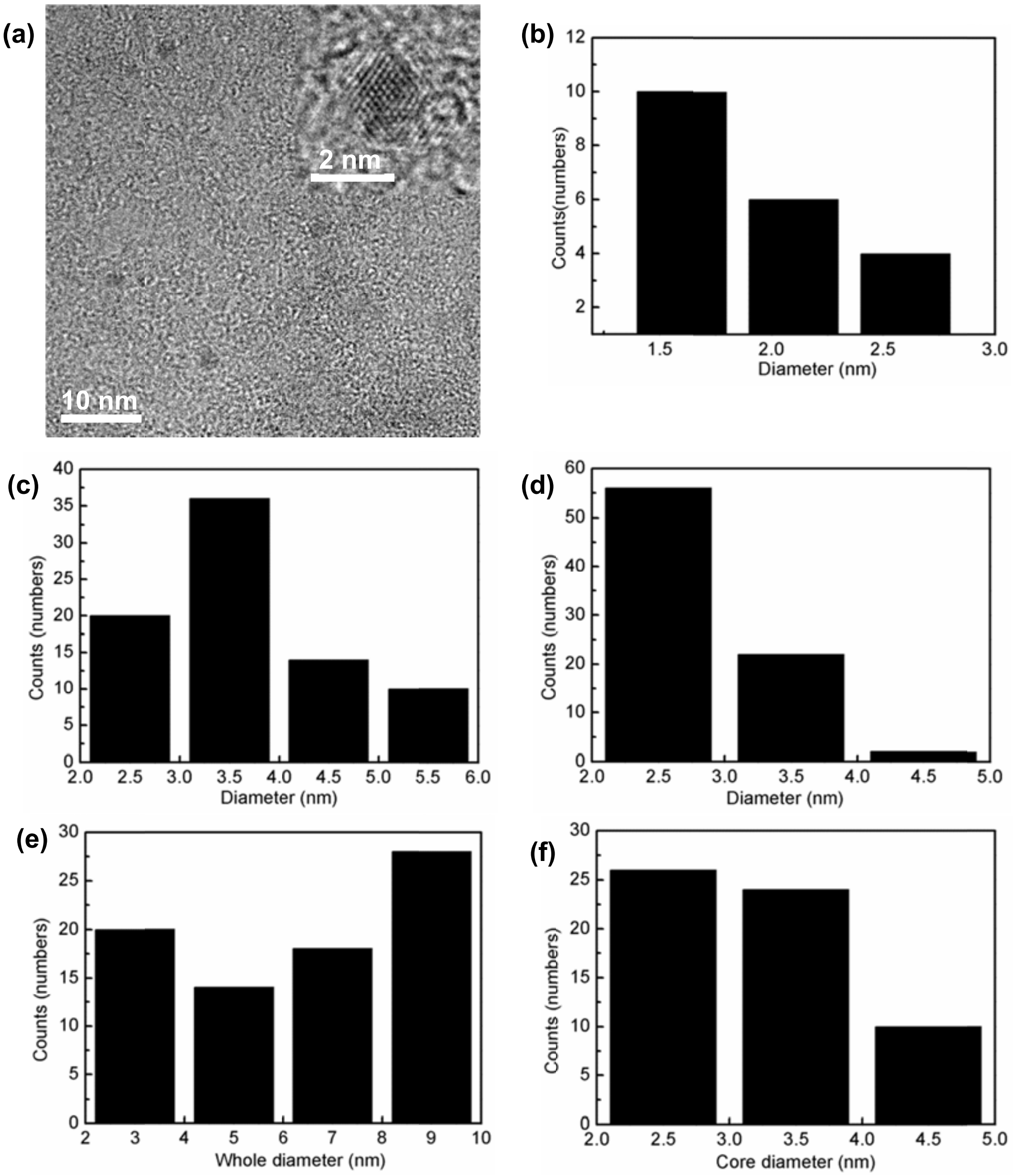

Figure s2 (a) TEM image of the rarely observed nanoparticles in the white intermediate solution that is formed as the reaction proceeds about 0.39 s (L_{total} = 10 cm) and (b), (c), (d), (e) and (f) the histogram of size distribution of nanoparticles formed during TEM sample preparation in the white intermediate solution, of nanoparticles in the reaction solutions after the reaction proceeds 0.44 s (L_{total} = 15 cm for Figure 1b), 1.03 s (L_{total} = 35 cm for Figure 1c and 1d) and 1.77 s (L_{total} = 60 cm for Figure 1d), respectively.

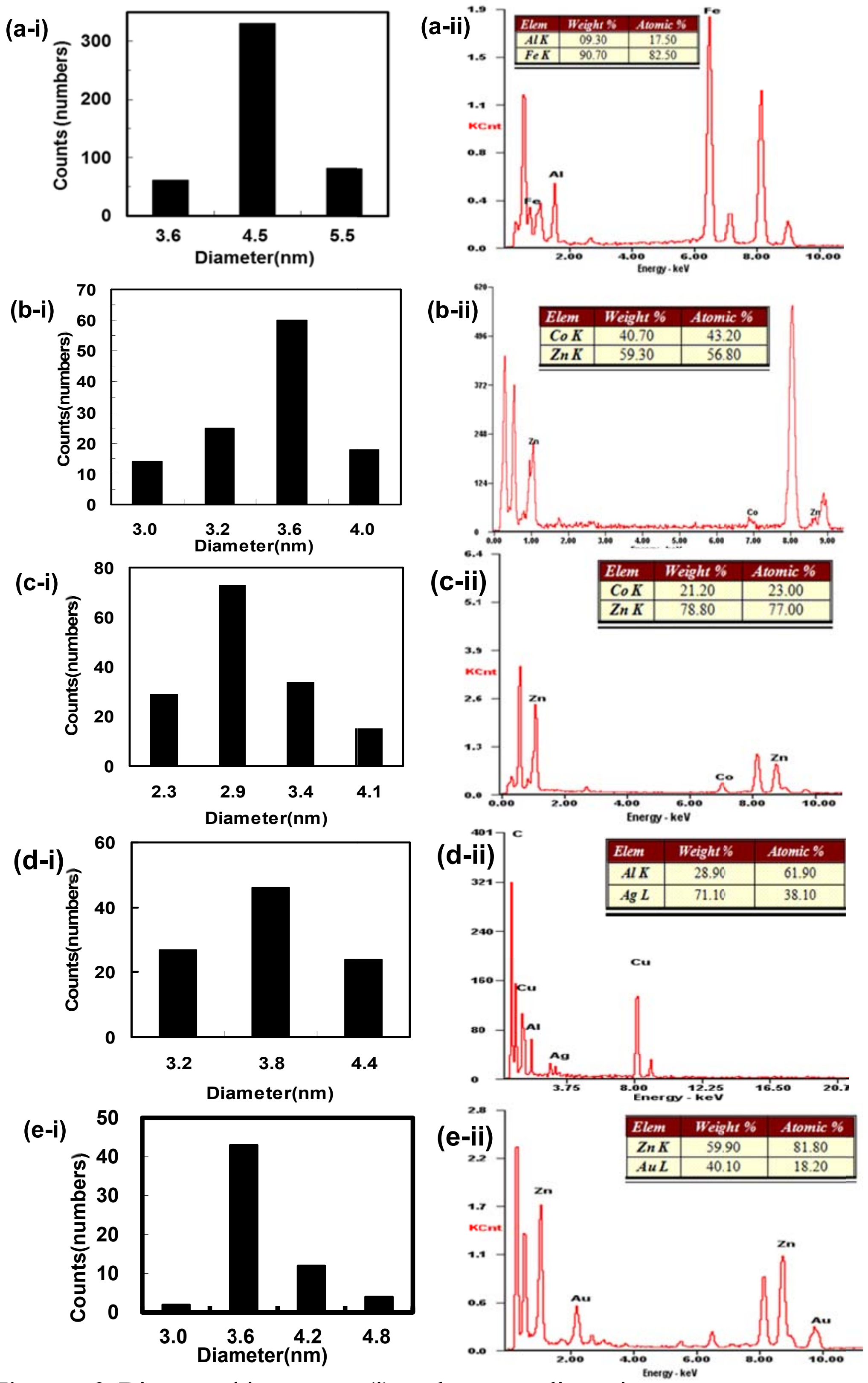

Figure s3 Diameter histograms (i) and energy dispersive x-ray spectroscopies (EDX) (ii) of (a) FeAl@$Al_{(1-x)}Fe_xO_y$ NPs and (b) CoZn@$Zn_{(1-x)}Co_xO_y$ NPs synthesized using all-NMP-phased process, and (c) CoZn@$Zn_{(1-x)}Co_xO_y$ NPs, (d) AgAl@$Al_{(1-x)}Ag_xO_y$ NPs and (e) AuZn@$Zn_{(1-x)}Au_xO_y$ NPs synthesized using aqueous-phased metal salt reaction systems.

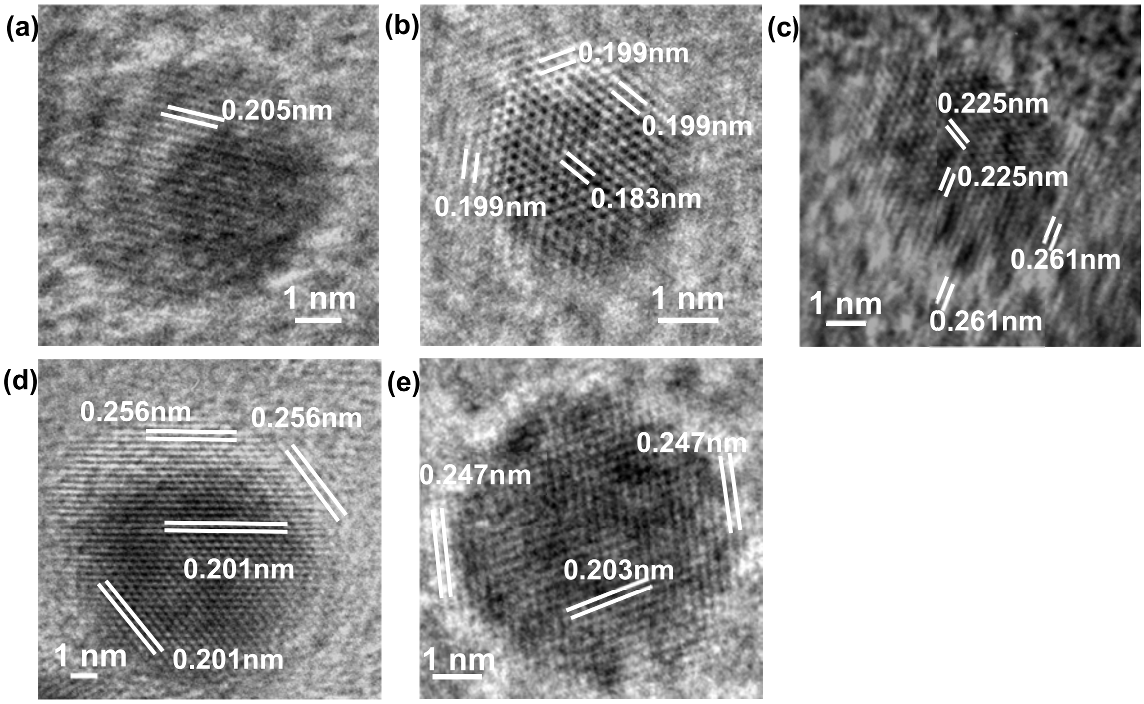

Figure s4 HR-TEM images with measured crystal lattices in cores and surface layers of (a) FeAl@$Al_{(1-x)}Fe_xO_3$, (b) CoZn@$Zn_{(1-x)}Co_xO_y$ NPs synthesized by all NMP-phased reaction systems; (c) CoZn@$Zn_{(1-x)}Co_xO_y$, (d)AgAl@$Al_{(1-x)}Ag_xO_y$ and (e) AuZn@$Zn_{(1-x)}A_xO$ nanohybrids synthesized using aqueous-phased metal salt reaction systems**.** HR-TEM images show different crystal lattices between alloy cores and surface coatings (Fe, Co, Ag or Au doping ZnO or Al_2O_3).

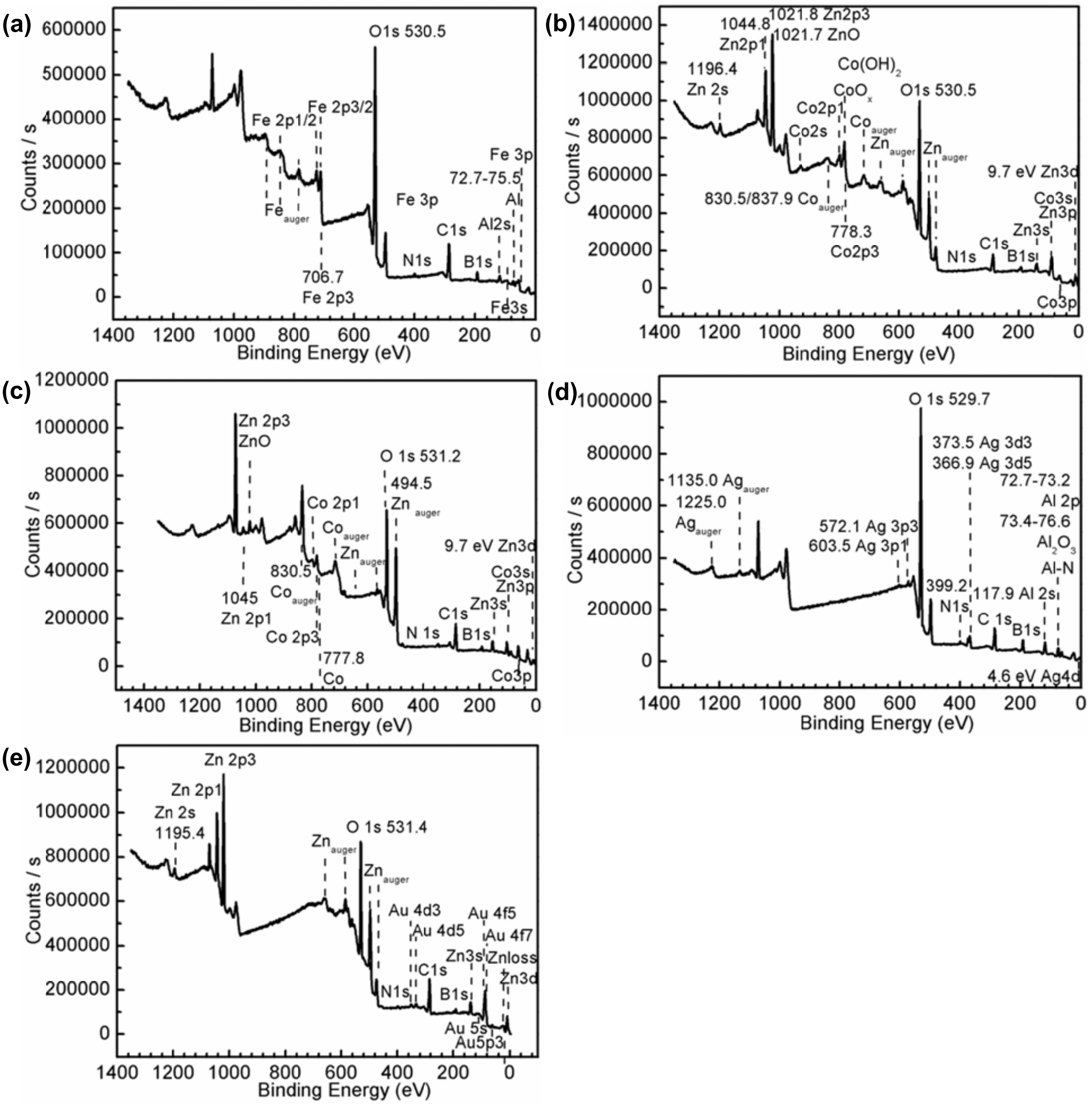

Figure s5 Full range X-ray photoelectron spectroscopy (XPS) of (a) $FeAl@Al_{(1-x)}Fe_xO_y$ and (b) $CoZn@Zn_{(1-x)}Co_xO_y$ NPs synthesized by all NMP-phased reaction systems; and (c) $CoZn@Zn_{(1-x)}Co_xO_y$, (d) $AgAl@Al_{(1-x)}Ag_xO_y$ and (e) $AuZn@Zn_{(1-x)}Au_xO_y$ nanohybrids synthesized using aqueous-phased metal slat reaction systems.

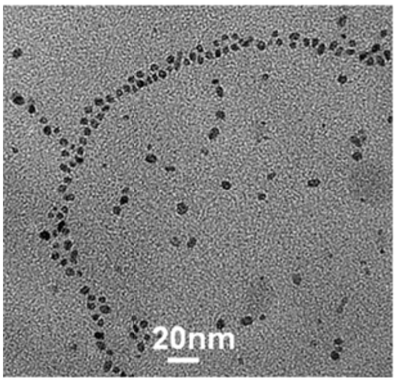

Figure s6 Chain-like nanoparticle assembly often observed in the re-dispersed $CoZn@Zn_{(1-x)}Co_xO_y$ NPs synthesized by aqueous-phased metal salt reaction system.

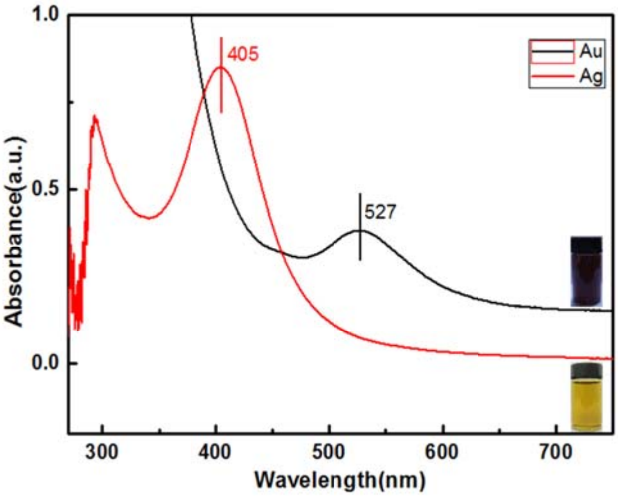

Figure s7 UV-vis spectra of 2.5 ± 0.2 nm Au and 3.5 ± 0.3 nm Ag nanoparticles synthesized by aqueous-phased metal salt reaction system.

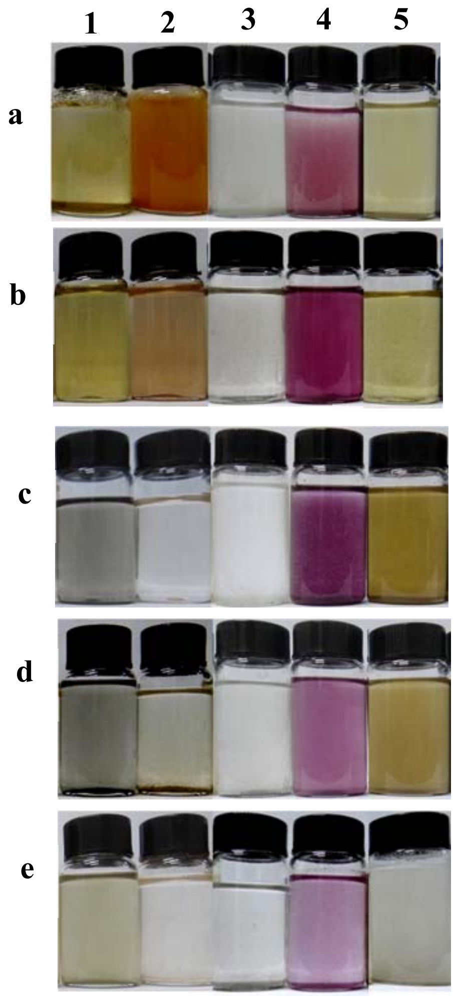

Figure s8 Solution color of the nanohybrids of (1) CoZn-$Zn_{(1-x)}Co_xO$ and (2) FeAl-$Al_{(2-x)}Fe_xO_3$ nanohybrids synthesized by all NMP-phased reaction systems, and (3) CoZn-$Zn_{(1-x)}Co_xO$, (4) AuZn@$Zn_{(1-x)}Au_xO$ and (5) AgAl@$Al_{(2-x)}Ag_xO_3$ nanohybrids synthesized by aqueous-phased nanohybrids in polar and non-polar solvents by directly re-dispersing their dry powders. Solvent types: (a) Water; (b) N-methyl-pyrrolidone (NMP); (c) Chlorobenzene; (d) Chloroform; (e) Cyclohexane. Solubility of these NPs are listed in Table 4

References

1 Gravesen, P., Branebjerg, J. & Jensen, O. S. Microfluidics-a review. *J. Mioromech. Microeng.* **3**, 168-182 (1993).

2 Song, Y., Kumar, C. S. S. R. & Hormes, J. Fabrication of SU-8 based microfluidic reactor on a PEEK substrate sealed by a "flexible semi-solid transfer'(FST) process. *J. Micromech. Microeng.* **14**, 932-940 (2004).
